# Resolving the conflicts around Par2 opposing roles in regeneration by comparing immune-mediated and toxic-induced injuries

**DOI:** 10.1186/s41232-022-00238-2

**Published:** 2022-11-29

**Authors:** Gal Reches, Netta R. Blondheim Shraga, Florent Carrette, Assaf Malka, Natalia Saleev, Yehuda Gubbay, Offir Ertracht, Izhak Haviv, Linda M. Bradley, Fred Levine, Ron Piran

**Affiliations:** 1 The Azrieli Faculty of Medicine, Bar-Ilan University, 8 Henrietta Szold St, Safed, Israel; 2 Eliachar Research Laboratory, Galilee Medical Center, Nahariya, Israel; 3 Infectious and Inflammatory Disease Center, Sanford Burnham Prebys Medical Discovery Institute, La Jolla, California, USA; 4 Sanford Children’s Health Research Center, Sanford Burnham Prebys Medical Discovery Institute, 10901 N Torrey Pines Rd, La Jolla, CA 92037 USA

**Keywords:** Protease-activated receptor-2 (Par2), Hepatitis, Liver regeneration, Concanavalin A, Carbon tetrachloride

## Abstract

**Background:**

Different factors may lead to hepatitis. Among which are liver inflammation and poisoning. We chose two hepatitis models, typical for these two underlying causes. Thus, we aimed to characterize the role of protease-activated receptor 2 (Par2) in liver regeneration and inflammation to reconcile Par2 conflicting role in many damage models, which sometimes aggravates the induced damage and sometimes alleviates it.

**Methods:**

WT and knockout (Par2KO) mice were injected with concanavalin A (ConA) to induce immune-mediated hepatitis or with carbon tetrachloride (CCl_4_) to elicit direct hepatic damage. To distinguish the immune component from the liver regenerative response, we conducted bone marrow (BM) replacements of WT and Par2KO mice and repeated the damage models.

**Results:**

ConA injection caused limited damage in Par2KO mice livers, while in the WT mice severe damage followed by leukocyte infiltration was evident. Reciprocal BM replacement of WT and Par2KO showed that WT BM-reconstituted Par2KO mice displayed marked liver damage, while in Par2KO BM-reconstituted WT mice, the tissue was generally protected.

In the CCl_4_ direct damage model, hepatocytes regenerated in WT mice, whereas Par2KO mice failed to recover. Reciprocal BM replacement did not show significant differences in hepatic regeneration. In Par2KO mice, hepatitis was more apparent, while WT recovered regardless of the BM origin.

**Conclusions:**

We conclude that Par2 activation in the immune system aggravates hepatitis and that Par2 activation in the damaged tissue promotes liver regeneration. When we incorporate this finding and revisit the literature reports, we reconciled the conflicts surrounding Par2’s role in injury, recovery, and inflammation.

**Supplementary Information:**

The online version contains supplementary material available at 10.1186/s41232-022-00238-2.

## Background

Interpreting the different pathways by which cells and tissues sense and react to injury is vital for developing treatments to facilitate tissue regeneration [1. –3. ]. Moreover, the ability of tissues to recover from an injury is fundamental for organismal survival, but the molecular mechanisms by which cells sense and respond to injury remain poorly understood [4. , 5. ]. The protease-activated receptor-2 (Par2) is a member of the G-protein-coupled receptor (GPCR) family. It plays important roles in growth, differentiation, and response to injury [6. –9. ]. Par2 was found to participate in pain signaling [10. –13. ] and in a range of inflammatory processes [14. –17. ]. PARs activation is achieved by N-terminus proteolytic cleavage. Par2 activation is predominantly mediated by trypsin, which is secreted by many cell types, and by additional serine proteases, some of which are membrane-anchored, generated during injury and inflammation. For example, factor Xa was proven to activate Par2 and leads to TGF-β expression [18. ]. These events further activate signal-related kinases/mitogen-activated protein kinases and sensitize transient receptor potential vanilloid (TRPV) ion channels by mechanisms involving cAMP and cGMP [19. , 20. ]. These latter events increase Ca^2+^ in many cell types, including neurons, astrocytes, and tumor cells [21. –24. ]. The binding of β-arrestin to phosphorylated residues on the Par2 C-terminal tail uncouples and terminates G-protein signaling, thereby resulting in endocytosis of the complex and in termination of the signal [25. –27. ]. Par2 is also hypothesized to mediate COVID-19 gut infection via the Zonulin pathway [28. ].

Recently, many studies have been shedding more light on Par2’s molecular mechanism of action. It was shown that thrombin-activated Par2 generated cAMP, which prevented Ca^2+^ influx in the immune system [29. ]. In keratinocytes, Par2 was found to function upstream to TRPV3, again, via Ca^2+^ signaling [30. ]. Moreover, TRPV3 increase dramatically in the lesioned skin of Par2 overexpressing mice [31. ]. In primary human nasal epithelial cells and mice tracheal epithelial cells, Par2 was suggested to act together with TRPV1 [32. ]. Another study, conducted in sensory neurons and keratinocytes, demonstrated that Pacific ciguatoxin-2, a main food poisoning agent, activated a severe response by Ca^2+^ signaling [33. ]. In cerebral mast cells, Par2 was suggested to activate neuroinflammation through NFkB pathways [34. , 35. ]. In hepatocytes, FoxO6 was demonstrated to mediate inflammation and insulin resistance via Par2 [36. ].

While the biochemical pathways of Par2 signaling have been extensively studied, the activation of the same biochemical pathway in different cell types and tissues can initiate different cellular, as well as organismal phenotypes, demonstrating that in order to understand the contradictions observed in Par2’s phenotypes, experiments are required at the whole organism level.

We have previously shown that the activation of Par2 has a pivotal role in different regeneration models and that Par2 knockout (Par2KO) mice are unable to regenerate damaged tissues. Specifically, in the digit, we showed that amputation in the middle of the distal phalanx led to effective regeneration in WT but not in the Par2KO mice. In the pancreas, we demonstrated that WT mice completely recovered from caerulein-induced pancreatitis, while regeneration was not observed in Par2KO mice, which died within 18 days [37. ]. In the endocrine pancreas, we showed that *β*-cell regeneration was mediated by Par2, similar to its activity following the administration of alloxan plus caerulein [38. ]. In the liver, we showed that Par2’s role in regeneration was demonstrated in the classical carbon tetrachloride (CCl_4_) model of direct hepatic damage [37. ].

As described above, there is an ample amount of scientific reports on Par2’s central role in many damage models. However, one cannot draw a more solid conclusion concerning exactly when, how, and under which circumstances Par2’s modulation should occur. Par2’s role seems to be contradictory, sometimes aggravating and other times alleviating the damage. For example, Par2 activation induces colonic inflammation [14. ] and Par2-mediated ovalbumin inhalation induces an allergic reaction [39. , 40. ]. On the other hand, Par2 activation protected against myocardial ischemia-reperfusion [41. ], as well as aggravated epithelial inflammation [42. ]. In some cases, Par2’s dual roles even take place in the same tissue, under different conditions. For example, in pancreatitis, Par2-mediated exocrine secretion promotes clearance of the pancreatic duct and acini from trypsin, thereby preventing tissue damage [8. , 43. ]. On the other hand, in a retrograde infusion of bile salt-induced pancreatitis, Par2 deficiency is protective and inflammation is milder [44. ]. Our goal in the current study was to reconcile Par2’s seemingly contradictory roles, sometimes in the same organ, in order to allow for a modulation strategy to emerge and perhaps allow future studies to use it for pharmaceutical applicability.

There are different types of damage models. Some are direct—as in the case of an injury, while others act via exaggeration of inflammatory processes. We hypothesized that by separating these two modes of action, we would be able to solve the Par2 argument presented above. Thus, we compared autoimmune hepatitis to direct hepatic damage. Among the classical models of induced hepatic damage, both concanavalin A (ConA) and CCl_4_ are leading methodologies, which have been employed to study liver necrosis, fibrosis, and hepatitis [45. –47. ]. While both methods induce liver damage, there are fundamental differences in the etiology and the final apparent damage. Injection of the lectin ConA leads to hepatic inflammation and has been studied for many years as a model of autoimmune hepatitis. It is induced by T-lymphocyte-mediated hepatocellular damage, which closely mimics the pathogenesis mechanisms and pathological changes of autoimmune hepatitis [48. , 49. ]. When ConA is injected, it binds first to mannose receptors found on the surface of the liver sinusoid lining cells. Then, these cells internalize the receptor and present ConA on their major histocompatibility complex (MHC) molecules [50. ]. In addition, it was found that Kupffer cells as well as T cells can bind ConA in an independent manner [51. , 52. ]. Both pathways subsequently lead to lymphocyte-dependent hepatitis. However, in the CCl_4_ model, hepatocellular injury is directly generated as a result of free radical formation [37. , 53. , 54. ]. A single dose of injected CCl_4_ in oil induces increased liver weight, elevated fat levels, serum urea, liver enzyme activities, and clear histopathological evidence of liver damage with single-cell necrosis [37. , 55. ]. The hepatotoxicity generated by CCl_4_ also induces liver regeneration in a similar fashion to hepatectomy [56. ].

WT and Par2KO mice were injected with ConA for the (auto) immune model or with CCl_4_ for the direct hepatic damage. To separate the immune component, we took advantage of the fact that all the immune cells are of hematopoietic lineage and could thus be replaced by bone marrow (BM) transplantation. Using reciprocal BM replacement experiments, we show that Par2 activation in the immune compartment is required for the appearance of inflammatory infiltrates, and in the damaged tissue, it is required for the regeneration of the damaged hepatocytes. Thus, we found that Par2 function in alleviating vs. aggravating the disease phenotype is determined by the interplay between two major factors: the immune system and the affected tissue, depending on the dominance of the one that is more significant in the damage model selected. Comparing the models, we conclude that when Par2 is activated in the immune system, it aggravates inflammation, and when it is activated in the damaged tissue, it promotes regeneration.

## Methods

### Mice

C57/BL6, Par2KO (CD45.2), and CD45.1 mice (B6.Cg-F2rl1tm1Mslb/J and B6.SJL-*Ptprc*^*a*^ *Pepc*^*b*^/BoyJ, from Jackson labs, strains # 004993 and #002014, respectively, Bar Harbor, ME, USA) were under pathogen-free conditions in the animal facility of the Azrieli Faculty of Medicine, Bar-Ilan University, Safed, Israel. All animal experiments were conducted according to the institutional animal ethical committee guidelines (Permit Number: 91-11-2017), which conform to the Guide for the Care and Use of Laboratory Animals published by the US National Institutes of Health (Eighth edition 2011). The animals were maintained at the institutional Experimental Surgical Unit, fed on a normal rodent chow diet, with tap water ad libitum. The mice were housed at a constant temperature and relative humidity under a regular light/dark schedule (12:12). Males and females were used equally in all experiments.

### Hepatitis induction administration

#### Inflammation model

WT and Par2KO mice were injected with a single IV injection of 10 mg/kg ConA (Sigma-Aldrich, St. Louis, MO, USA) in phosphate-buffered saline (PBS, Biological Industries, Beit-Haemek, Israel). Mice were sacrificed 1 or 14 days after injection (3–4 mice were in each group). For high-dose ConA treatment, WT and Par2KO mice were injected with a single IV injection of 15mg/kg ConA. In the high-dose experiment, mice were sacrificed 6 h after injection. Blood samples were taken, and the livers were harvested and analyzed by histochemical and immunohistochemical staining (7–12 mice were in each group).

#### Direct toxicity model

WT and Par2KO mice were injected with a single IP injection of 1 ml/kg (1.594 g/kg) CCl_4_ (Sigma-Aldrich, St. Louis, MO, USA, 10% solution in olive oil (Yad-Mordechai, Israel). Mice were sacrificed 1 day or 7 days after injection. Blood samples were taken, and the livers were harvested and analyzed by histochemical staining (3–7 mice were in each group).

#### Liver enzyme analysis

Serum from experimental mice was collected and analyzed by a VetScan VS2 (Abaxis Veterinary Diagnostics, Union City, CA, USA) at the Sanford Burnham Prebys Medical Discovery Institute animal core facility (La Jolla, CA, USA).

#### Immuno and histochemical staining

The livers were fixed in formaldehyde (4% in PBS, Santa Cruz Biotechnology, Dallas, TX, USA). The livers were embedded in paraffin, sectioned into 5μm using a microtome, and loaded onto microscope glass slides at the Nahariya Galilee Medical Center’s research unit. Hematoxylin and eosin (H&E) as well as Picro-Sirius Red staining were performed. After de-paraffinization and re-hydration, slides were rinsed in distilled water and stained for nuclei with Weigert’s hematoxylin solution for 5 min. Then, the slides were placed in Picro-Sirius red stain for 60 min, then rinsed twice in 0.5% of the acetic acid solution, followed by dehydration quickly through 3 changes to absolute ethanol. The slides were scanned at a magnification of 20×, and images were acquired by the Axio Scan.Z1 microscope (Zeiss, Jena, Germany).

#### Immunohistochemical staining

The slides were washed three times with PBS and treated with 0.3% Triton X-100 (MP Biomedicals, Solon, OH, USA) for 15 min, then washed with PBS for 10 min. The slides were incubated in a blocking solution with 5% normal donkey serum (Jackson ImmunoResearch, West Grove, PA, USA) for 50 min at room temperature (RT). The slides were incubated with antisera specific for Par2 (1/400, goat, sc-8207, Santa Cruz Biotechnology). HRP-conjugated secondary antibodies (Jackson ImmunoResearch) were used to visually label Par2 localization (DAKO, Carpinteria, CA, USA). The slides were scanned at a magnification of 20× using the Aperio Scanscope FL system (Aperio Technologies Inc., Vista, CA, USA).

#### Immunofluoroscence staining

The slides were incubated overnight at 4° C with primary antibodies for Par2 (1/200, mouse, Thermos Fisher Scientific, San Diego, CA, USA), CD45 (1/200, rat, Novus Biological, Centennial, CO 80112, USA), and albumin (1/100, gout, R&D Systems Inc., Minneapolis, MN, USA). Secondary antibodies were labeled with Alexa Fluor 488, Alexa Fluor 647, and Rhodamine Red (all from Jackson ImmunoResearch). Nuclei were visualized with DAPI Fluoromount-G^TM^ (Bar Naor Ltd, Ramat Gan, Israel). The slides were imaged using Microscope Axio Imager M2 (Zeiss).

#### Cell death identification—TUNEL staining

To visualize cell death, TUNEL staining was performed with TUNEL Andy Flou^TM^ 594 Apoptosis detection kit (ABP Biosciences, Beltsville, MD, USA) as described in their protocol. The slides were scanned at a magnification of 20×, and images were acquired by the Axio Scan.Z1 microscope (Zeiss). For analysis, rectangles of 1,000,000μm^2^ were analyzed by ImageJ for TUNEL positive area (594nm).

#### Quantification of liver damage

For the ConA experiments, selected areas of the slides were chosen for figures using Aperio Imagescope (version 12 Aperio Technologies Inc.). For analysis, the slide areas were selected and analyzed using the web-based Image Scope viewer. Nuclear density was measured by creating a rectangle of 100μm^2^ surrounding Par2 positive or negative areas. Necrosis was identified as a lack of nuclear staining in H&E (thus creating pink-distinctive staining).

For the CCl_4_ experiments, the liver damage was measured using the ZEN software (Zeiss). Damage was measured as the number of fat vacuoles with an area of 25,000μm^2^ (day 1) or as the percentage area of necrotized lesions in a macroscopic picture (day 7).

#### Irradiation and bone marrow chimeras

To generate WT hosts recipients of Par2KO BM, 6-week-old WT (CD45.1), mice were lethally irradiated (1000 Rad) in their cages at the Rivka Ziv’s hospital radiology unit (Safed, Israel) and reconstituted with BM isolated from 9-week-old Par2KO donor mice (CD45.2). To generate Par2KO hosts of WT BM, 6-week-old Par2KO mice (CD45.2) were irradiated and reconstituted with BM isolated from 9-week-old WT donor mice (CD45.1) as described above.

As controls, CD45.1 WT host recipients were reconstituted with BM isolated from CD45.2 WT donors as described above. Donor mice were sacrificed by isoflurane overdose, and BM cells were collected from femurs and tibias. BM cells were pooled together and centrifuged at 600 G for 10 min. The supernatant was discarded, and the pellet was resuspended in saline calculated as 100μl per recipient mouse [=100*(*n*+2)]. Four hours post-irradiation the recipient mice were reconstituted with donor BM by IV injections. Mice were treated with antibiotics [3.3ml of Septrin (40mg/ml sulfamethoxazole and 8mg/ml trimethoprim, Teva Pharmaceutical Industries Ltd., Petah Tikva, Israel) in 250ml drinking water for 3 weeks]. FACS analysis was conducted for the BM replacement validation: 3 weeks after BM replacement, the blood samples were taken from the mice cheek, 50μl blood from each mouse was washed in 1ml PBS/2%FBS (Biological Industries) and centrifuged at 1200 rpm for 5 min. Antibodies were added according to table S1.

Cells were incubated for 40 min in RT. One milliliter of red blood cells (RBC) lysis buffer (Biological Industries) was added, then tubes were incubated for 10 min in RT and were washed in 1ml PBS/2%FBS, centrifuged at 1200 rpm for 5 min. 150ml of FBS was added, and the contents were poured into the FACS tubes and loaded into the Gallios FACS analyzer (Beckman Coulter Life Sciences, Indianapolis, IN, USA). CD4 and CD8 T cells were gated on CD45.1 for WT donor into Par2KO and on CD45.2 for the reciprocal BM replacement. Three weeks post-BM reconstitution, hepatitis induction administration for the two injury models was carried out as described before.

#### T cell depletion

WT and Par2KO mice were IP injected with 500μg of anti-CD4 and 500μg of anti-CD8 antibodies (Bio X Cell, Lebanon, NH, USA), 2 days prior and 1 day prior to ConA treatment. FACS analysis was conducted to validate the depletion of CD8 and CD4 T cells, as described above in the BM validation section.

### Isolation and characterization of liver immune cells

The enzymatic digestion method was applied for the isolation of intrahepatic lymphocytes by modifying a previously described protocol [57. ]. Perfusion buffer (PB) was prepared by diluting 40ml of perfusion buffer concentrate [500ml PBC= 3.55 M NaCl, 168mM KCl (Sigma-Aldrich), 240mM HEPES (Biological Industries), 150mM NaOH (Sigma-Aldrich), in distilled deionized H_2_O] into 960-mL ultrapure H_2_O. A pre-warmed syringe was filled with 50ml of PB at 37°C.

Fifty milliliters of dissociation buffer was prepared by 49.5ml PB supplemented with 0.5ml 476mM CaCl_2_ (Sigma-Aldrich) and 3600U Collagenase Type IV (Worthington Biochemical Corporation, Lakewood, NJ, USA). Pre-warmed syringe was filled with 50mL of dissociation buffer at 37°C.

Mice were euthanized by isoflurane overdose. Suprahepatic inferior vena cava (IVC) was clamped to maintain localized perfusion, and a 23-G needle attached to a syringe filled with pre-warmed PB was gently inserted into the subhepatic IVC. The portal vein was cut to allow PB and blood flow through the liver. The liver was perfused until blood was no longer visible. PB syringe was replaced with the pre-warmed dissociation buffer syringe, and the liver was perfused until it was fully digested. The liver was carefully removed from the mouse to a 10ml ice-cold DMEM (Biological Industries). Then, the Petri dish was gently dispersed using forceps. Saturated DMEM was passed through a 70μm cell strainer (Jet Biofil, Guangzhou, China) and attached to a 50ml collection tube. Cell suspension was centrifuged at 50G for 2 min at 4°C, and the supernatant was collected. This step was repeated 2 more times. Then, the supernatant was centrifuged at 300G for 10 min at 4°C. The cell pellet was washed in PBS/2%FBS. Samples were incubated with FcR Blocking Reagent (Miltenyi Biotec, Auburn, CA, USA) for 10 min at 4°C before being stained with monoclonal antibodies for an additional 20 min at 4°C according to table S1. Cells were washed in 1ml PBS/2%FBS and centrifuged at 1200 rpm for 5 min. 150μl PBS/2%FBS was added, and the contents were transferred into the FACS tubes (1μl of each antibody was added to 1×10^6^ cells in 100μl PBS/2%FBS). Cells were analyzed on a Gallios flow cytometer, and at least 20,000 gated events were analyzed using Kaluza software (Beckman Coulter).

### Statistical analysis

Statistical analysis was carried out using GraphPad Prism 5.0, evaluating the differences between different groups. All data were presented as mean ± SEM. Comparisons between groups for statistical significance were performed with Student’s *T* test or Mann–Whitney test. Survival curves among groups were assessed by Mantel-Cox log-rank test. Results were considered significant difference at *P < 0.05, **P < 0.01, ***P < 0.005.

## Results

### Par2 aggravates inflammation as demonstrated by ConA-induced hepatitis

**Fig. 1:**
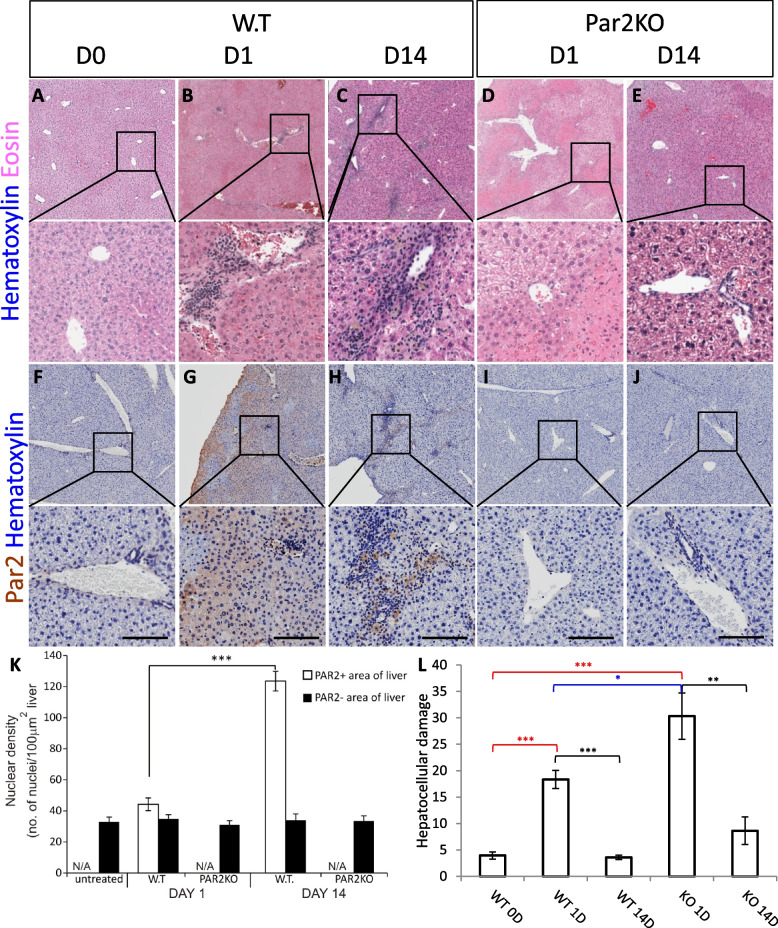
Mononuclear cell infiltrates induced by ConA are decreased in Par2KO compared to WT mice. One day after injection, WT mice treated with ConA (10mg/kg) had areas of hemorrhage and mononuclear cell infiltrates (compare **B**, **G** to untreated mice in **A**, **F**). These infiltrates were less severe in the Par2KO (compare **D**, **I** to **B**, **G**), although specifically, hepatocellular necrosis is more apparent in Par2KO (**D**). In the WT, mononuclear cell infiltrates were more evident at day 14 (**C**, **H**), while in the Par2KO, there were no infiltrates (**D**, **E**, **I**, **J**). Scale bar = 75μm. **K** Quantification of mononuclear cell infiltrate in Par2-positive and negative areas. **L** Quantification of the hepatocellular damage (the percentage area of necrotic lesions) presented in **A**–**J** (*n*=4 for each condition). Note that hepatocellular damage is observed both in WT and Par2KO regardless of leukocyte infiltration. Black—statistical significance within the same group. Red—statistical significance in comparison to untreated WT. Blue—statistical significance between WT and KO at day 1. Statistical significance was measured using *T* test. *Indicates *p*<0.05, **p<0.01, ***p<0.005, error bars = SEM

**Fig. 2:**
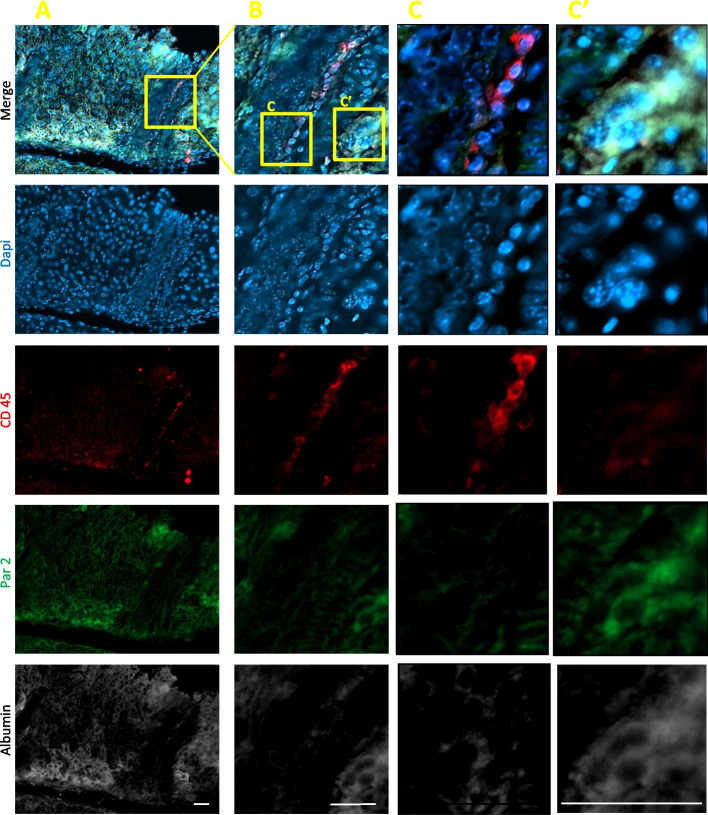
Par2 expression is maintained in WT hepatocytes and not in WT leukocytes 14 days after ConA injection. The core of large infiltrates is composed of apoptotic cells, which are CD45, Par2, and albumin negative. The apoptosis is indicated by the loss of the normal nuclei structure (note the ring-like circle of the Dapi staining, most apparent in **C**. The outer layer of the mononuclear infiltrate is composed of CD45+ leukocytes (shown in red). At day 14, these cells do not express Par2 and present normal nucleus. Healthy hepatocytes are not present in the infiltrate and observed by the uneven albumin staining of the infiltrate. At this stage of the ConA model, all apparent Par2+ cells express albumin (most observed in **A** and **C**’). The albumin+ staining is an indication of a functional hepatocyte. **A** Low, **B** middle, and **C** high magnifications. Scale bar = 50μm

Recently, we showed that Par2 signaling is required for tissue regeneration and healing. Specifically, no liver regeneration was detected in Par2KO mice following CCl_4_ injection [37. ]. Par2KO mice develop normally, sharing the same developmental timeline as the WT [37. ]. Particularly for the liver, both macroscopically and microscopically, the WT and Par2KO livers appear normal (Figure S1). To test our hypothesis that Par2’s role in regeneration is dependent on the tissue in which Par2 is activated, we tested ConA-induced hepatitis in WT and Par2KO mice. When mice were injected with ConA, within 1 day, there was a rapid induction of Par2 expression in the WT liver from a low baseline level (Fig. 1F–G, higher magnification in Figure S2A), similar to what we showed previously with CCl_4_ [37. ]. At that early time, mononuclear cell infiltrations, the hallmark of autoimmune hepatitis [48. , 49. ], were detectable (Fig. 1B, G, quantified in K). The expression of inflammatory markers could not explain the difference in phenotypes as no alteration was observed between the sera of the different experimental groups (Figure S3). By day 14 in the WT, hepatic Par2 transient expression decreased to baseline in most areas of the liver, but immune mononuclear infiltration had become more extensive (Fig. 1C, H, quantified in K, larger field of view in Figure S2B). Strikingly, the mononuclear infiltrating cells in the liver were localized to regions that retained Par2 expression (Fig. 1H, Figure S2B, quantified in 1K). At this progressive stage (day 14), Par2 is expressed in hepatocytes adjacent to or in the inflammatory regions. Moreover, in the WT, Par2 co-localizes with albumin staining, and leukocytes do not express albumin, indicating that the infiltrating immune cells are responsible for maintaining hepatocellular Par2 expression (Fig. 2 and S4). In contrast, even though there were no mononuclear infiltrates in the livers of Par2KO mice (Fig. 1D, E, I, J), areas of necrosis were developed, a finding indicating that ConA *is* toxic to Par2KO hepatocytes *regardless* of leukocyte infiltration (Fig. 1D, quantified in 1L). This observation highlights a potential Par2 protective role in the WT hepatocyte, similarly to the protective role we found in pancreatic *β* cells [37. ].

#### Par2 has an aggravating role in immune stimulation

**Fig. 3:**
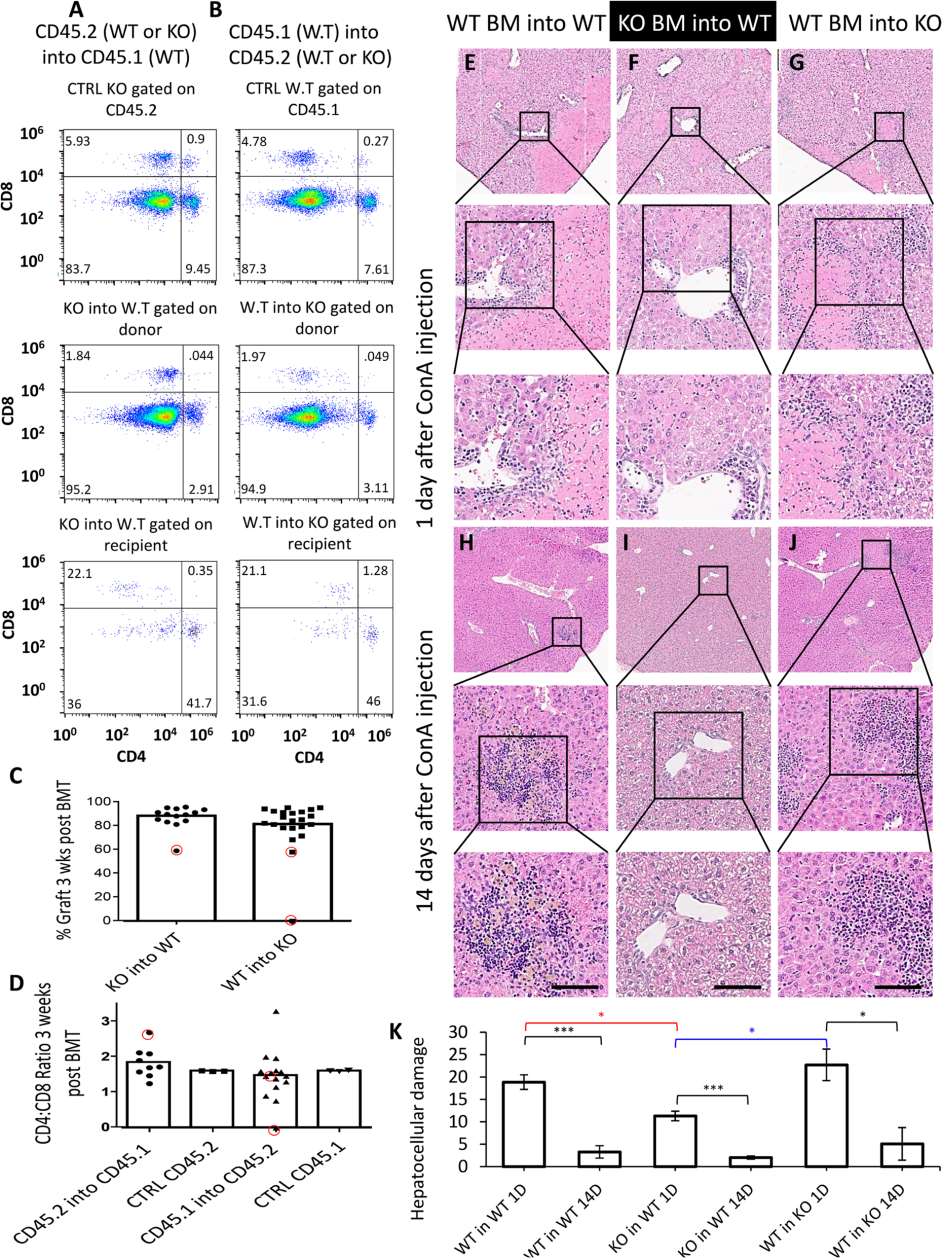
ConA-induced hepatitis is controlled by Par2 activation in the immune system. **A**, **C**, and **D** FACS analysis of CD45.2 mice (WT and Par2KO) that were used as BM donors to reconstitute WT CD45.1 mice, indicate that T-lymphocyte distribution after BM replacement was comparable in both setups, as both CD4 and CD8 T cells comprised an average of 88% of the donor T cells (**C**), *n*=14. **B**, **C**, and **D**. The reciprocal experiment: FACS analysis of WT CD45.1 that were used as BM donor to CD45.2 (WT and Par2KO) recipient mice. T cell distribution after BM replacement remained the same in both setups. The donor cells comprise ~81% of the T cells, *n*=22. Red circles indicate mice that were excluded from the experiment due to unsuccessful BM replacement (*n*=3). **E**, **H** A control experiment WT BM-reconstituted WT mice (CD45.1 into CD45.2) displays both mononuclear cell infiltration and hepatocellular damage. Note that hepatocellular damage is apparent on day 1 (**E**). **F**, **I** Par2KO BM-reconstituted WT mice show the relatively diminished levels of infiltrates. Note that hepatocellular damage is still apparent. **G**, **J** WT BM-reconstituted Par2KO mice. Infiltrates and tissue damage are apparent, indicating that mononuclear infiltrations are mediated via Par2 on immune cells and not via Par2 signaling in the hepatic tissue. Scale bar =75μm. **K** Quantification of the hepatocellular damage (the percentage area of necrotic lesions) presented in **E**–**J** (*n*=18, 3 for each group). Black—statistical significance within the same group. Red—statistical significance in comparison to control at day 1. Blue—statistical significance between the two reciprocal BM replacements. Statistical significance was measured using T test. *Indicates *p*<0.05, **p<0.01, ***p<0.005, error bars = SEM

To test the hypothesis that Par2 has two seemingly contradictory roles in injury and damage, depending if it is being activated in the immune system or the affected tissue, we performed reciprocal BM transplantations between WT and Par2KO mice (Fig. 3A, B, quantified in C, D), creating WT mice with Par2KO immune cells and Par2KO mice with WT immune cells. In Par2KO, BM-reconstituted WT chimeras, and ConA induced milder hepatitis, as demonstrated by lower mononuclear cell infiltrates and less hepatocellular damage (compare Fig. 3E to F, quantified in K). By day 14, the damage was completely resolved (Fig. 3I, quantified in K), resembling the complete Par2KO phenotype (Fig. 1E). The phenotype in WT BM-reconstituted Par2KO mice resembled the WT phenotype on day 1 (Fig. 1B, G, and the WT BM-reconstituted WT control—Fig. 3E), and the infiltrate similarity was also consistent on day 14 (compare Fig. 3J to H and Fig. 1C, H). Therefore, we conclude that in autoimmunity, leukocyte Par2 is propagating the inflammatory response induced by the immune system. These findings indicate that Par2 expression in hepatocytes is not required for immune cell infiltration of the liver. In fact, we did not see a difference in CD45+ cells between WT and WT BM-reconstituted Par2KO livers (data not shown). Thus, the mononuclear cells must be maintaining Par2 expression in the WT hepatocytes. Moreover, the fact that mononuclear infiltrations are sustained in WT BM-reconstituted Par2KO mice at day 14 indicates that the maintained Par2 expression in hepatocytes at this late stage is an inflammatory effect rather than a cause (Fig. 2 and S4).

#### Par2 in T cells is the key mediator for ConA-induced hepatitis, while its activation in hepatocytes has a protective role

**Fig. 4:**
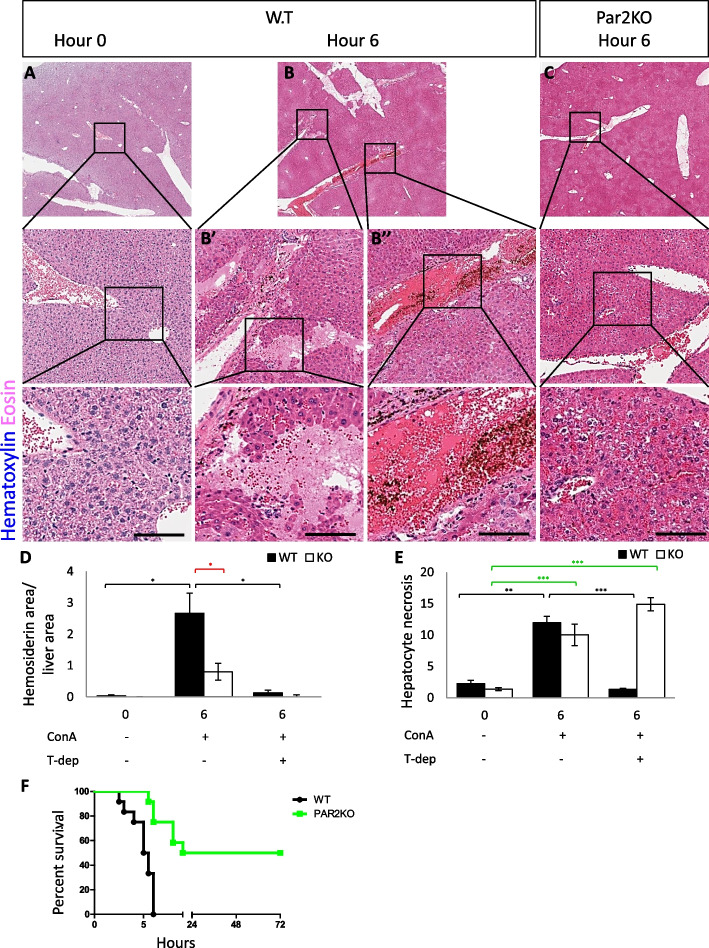
Par2KO improves survival from high-dose ConA-induced hepatitis. High-dose ConA (15mg/kg) induced acute liver lesions in all mice by 6 h after injection (**A**–**C**). Extensive hemorrhage was present in WT (**B**), but not in Par2KO (**C**) mice, 6 h after injection of ConA. Scale bar for **A**–**C** = 75μm. **D**, **E** T cell depletion rescued WT liver from damage—quantification of the data presented in supplemental fig 7. **D** Liver hemosiderin area was significantly greater in WT than in Par2KO mice 6 h after high-dose ConA injection (15 mg/kg, *n*=24, *n*=4 for each group). Following T cell depletion, there was no hemosiderin in the KO and almost none in the WT mice. **E** Hepatocyte necrosis appeared both in WT and Par2KO mice 6 h after high dose of ConA injection. T cell depletion diminished hepatocyte necrosis in the WT mice, while in the KO necrosis remained similar. Statistical significance was measured using T test. *Indicates *p*<0.05, **p<0.01, ***p<0.005, error bars = SEM. Black—between the WT groups; Red—in **D**, hemosiderin differences between WT and KO 6 h after ConA injection without T cell depletion. Green—in **E**, between the Par2KO groups. **F** Kaplan-Meier survival plot of WT (black, *n*=12) and Par2KO (green, *n*=12) after 15mg/kg ConA injection. Log-rank (Mantel-Cox) test for *p* value = 0.0004

**Fig. 5:**
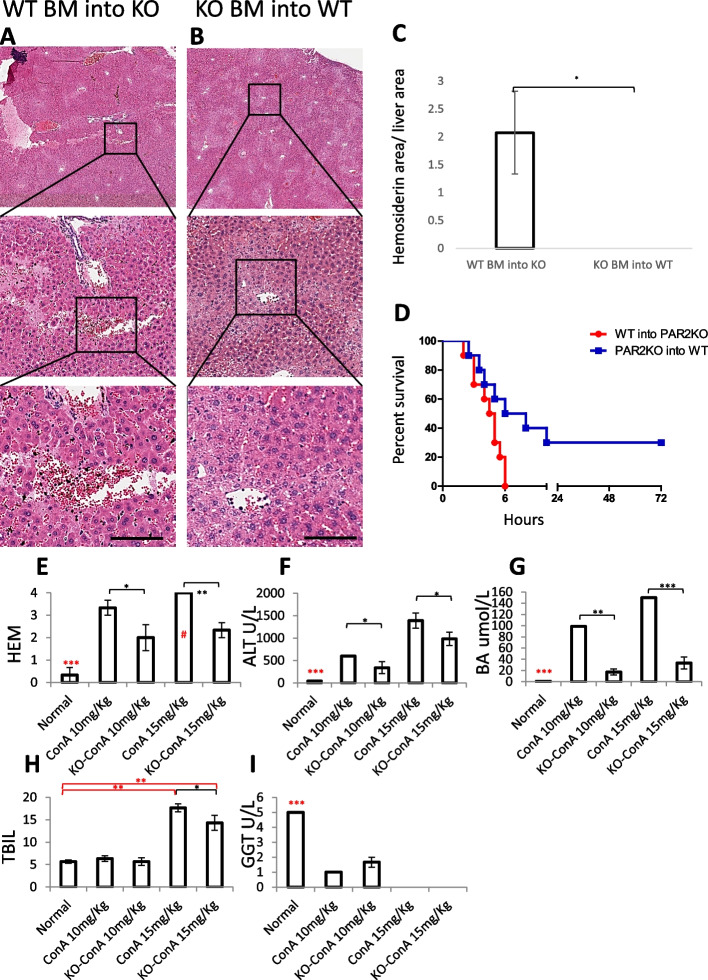
BM replacement experiment with high-dose ConA treatment show that the ConA effect is mediated by Par2 activation. **A** WT BM-reconstituted Par2KO mice 6 h after high-dose ConA injection showing hemorrhage. **B** Par2KO BM-reconstituted WT mice 6 h after high-dose ConA injection—hemorrhage was practically absent. Scale bar = 75μm. **C** Hemorrhage quantification (by hemosiderin area) shows that it appears in WT BM-reconstituted Par2KO mice and not in the reciprocal experiment (*n*=3 for each group). Statistical significance was measured using T test; error bars= SEM. **D** Kaplan-Meier survival plot of WT BM-reconstituted Par2KO mice (red, *n*=10) and Par2KO BM-reconstituted WT mice (blue, *n*=10) after 15mg/kg ConA injection. Log-rank (Mantel-Cox) test for *p* value = 0.0295. **E**–**I** Analysis of serum markers of liver damage 6 h after injection of 10 or 15 mg/kg ConA, determined by VetScan VS2. **E** Hemolysis (HEM). Note that 4 is the assay limit (#). **F** Alanine aminotransferase (ALT). **G** Bile acid (BA). **H** Total bilirubin (TBIL). **I** Gamma glutamyl transferase (GGT), (*n*=15, 3 for each group). Statistical significance was measured using T test. *Indicates *p*<0.05, **p<0.01, ***p<0.005, error bars = SEM. Black—between groups as indicated. Red—untreated WT control in comparison to all other groups or as indicated

While the phenotype of low-dose ConA is leukocyte infiltrations, the phenotype of high-dose ConA is more severe [49. ]. Within 6 h after the ConA injection, all WT animals had died. However, more than 50% of the Par2KO mice had survived and fully recovered (Fig. 4F). High-dose ConA induced acute liver lesions in all mice within 6 h after injection, with extensive hemorrhage present in the WT but not in Par2KO mice (compare Fig. 4B to C, quantified in D). Hepatic necrosis, however, was evident in both WT and Par2KO mice (Fig. 4B, C, quantified in E). Liver injury indicators, measured by VetScan VS2, were also more significant in WT compared with Par2KO mice. This finding was more significant in the higher dose (Fig. 5E–I).

**Fig. 6:**
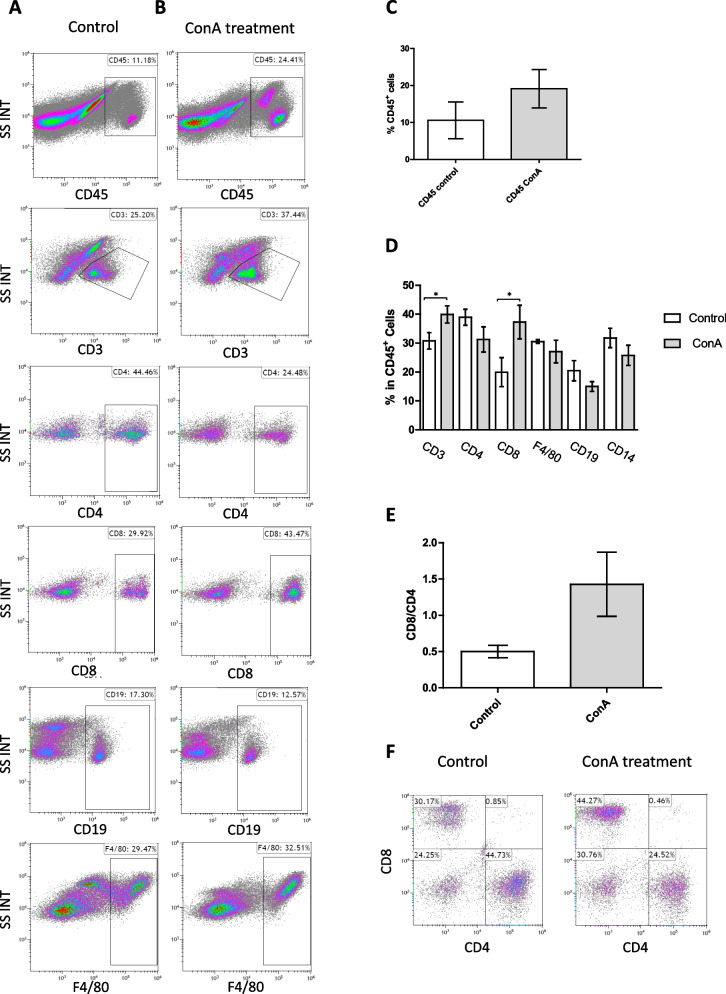
Immune cell characterization in the livers before and after ConA treatment. Fourteen days after ConA treatment, mice livers were characterized with higher T cells’ presence, from which, CD8-positive cells were increased while CD4-positive cells remained without a significant difference. **A**, **B** FACS analysis of different leucocytes extracted from the liver of control WT mice, *n*=3 (**A**) vs. ConA-treated WT mice, 14 days after ConA injection, *n*=5 (**B**). **C** The percentage of CD45-positive cells. **D** The percentage of co-expression of different immune markers gated on CD45-positive cells. **E** CD8/CD4 ratios gated on CD3 (an example of the analysis is in **F**). Statistical significance was measured using T test. *Indicates *p*<0.05, error bars = SEM

Previously, it was reported that ConA-induced hepatitis was the result of T cell activation [49. –51. ]. To validate this claim and to check whether other leukocytes like Kupffer cells and other macrophages participate in the infiltrates, we profiled the leukocytes in the perfused livers of WT ConA-treated mice. FACS analysis and immunostaining have indicated that significant changes were observed in the T cell population, which was significantly increased in the ConA-treated mice (CD3-positive cells in Fig. 6 and S5). We did not see an increase or proliferation of Kupffer cells and other macrophages in the infiltrates.

It was unclear if T cell-depleted animals were completely protected from ConA in a similar phenotype to the protection from ConA facilitated by Par2KO. To answer this question, we performed a T cell depletion assay in WT and Par2KO mice (Figs. S6 and S7). Mice were followed for 6 h after the injection. When T cells were depleted, the deleterious effect of ConA was abrogated in WT mice (Figure S7C). In the Par2KO mice, however, after T cell depletion, hemorrhage was completely absent. Yet, hepatocyte damage was still apparent (Figure S7D-E, quantified in Fig. 4D, E), proving that in addition to aggravating the ConA effect via T cells, Par2 has a protective role in hepatocytes.

To show that the lethal effect was mediated by the activation of Par2 in T cells, we repeated the BM replacement experiment with the high ConA dose (Fig. 5A, B, quantified in C). When WT BM was implanted into Par2KO mice, ConA led to hepatocellular damage and hemorrhage, and lethality was regained (Fig. 5A, C, D). When Par2KO BM was implanted into WT, ConA-mediated damage and lethality were diminished significantly (Fig. 5B–D), resembling the protection against ConA exhibited by the complete Par2KO.

#### Par2 is required for liver regeneration when direct damage is inflicted

**Fig. 7:**
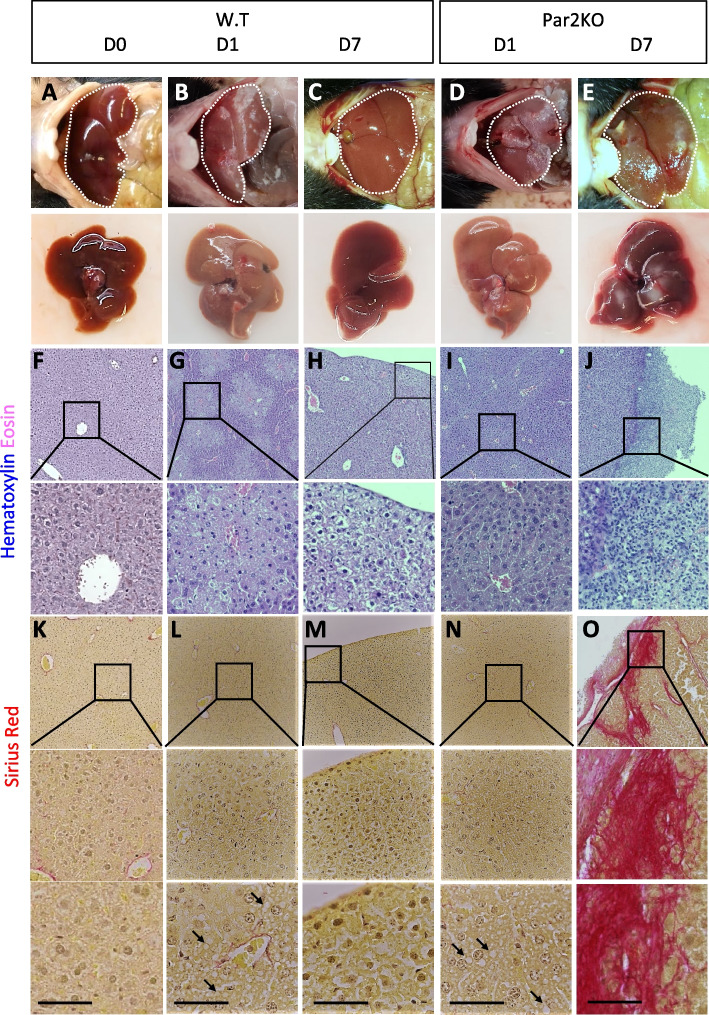
Par2 promotes regeneration in hepatocytes after CCl_4_ injection. WT mice recovered from the tissue damage caused by CCl_4_ while Par2KO mice failed to recover. **A**, **F**, and **K**. WT mouse before CCl_4_ injection. **B**, **G**, and **L**. WT 1 day after injection: fat vacuoles (arrows) indicate hepatic steatosis. **C**, **H**, and **M**. WT 7 days after injection: the tissue recovered. **D**, **I**, and **N**. Par2KO mice 1 day after CCl_4_ injection: fat vacuoles (arrows) indicate hepatic steatosis. **E**, **J**, and **O**. Par2KO mice 7 days after injection: necrotic lesions (E, J) and collagen fibers (O in pink) appeared. **A-E.** Macroscopic images. **F-J.** H&E, **K-O**. Sirius Red. Scale bar = 50μm

**Fig. 8:**
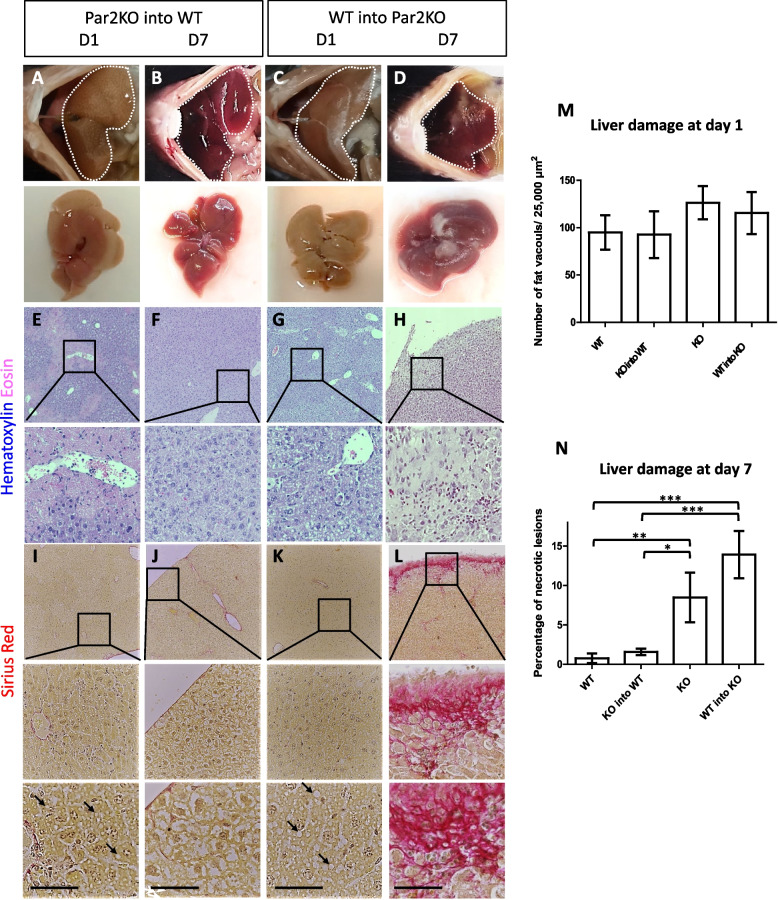
WT BM-reconstituted Par2KO mice exhibited apparent hepatitis, while in Par2KO BM-reconstituted WT mice recovered. **A**, **E**, and **I** Par2KO BM-reconstituted WT mice, 1 day after CCl_4_ injection: hepatic steatosis is apparent with fat vacuoles (arrows). **B**, **F**, and **J** Par2KO BM-reconstituted WT mice, 7 days after injection: hepatic tissue recovered. **C**, **G**, and **K** WT BM-reconstituted Par2KO mice, 1 day after CCl_4_ injection: hepatic steatosis is apparent with fat vacuoles (arrows, similarly to Par2KO BM-reconstituted WT group). **D**, **H**, and **L** WT BM-reconstituted Par2KO mice, 7 days after CCl_4_ injection: necrotic lesions (**D**, **H**) and collagen fibers (**L** in pink) appeared. **A**–**D** Macroscopic images. **E**–**H** H&E, **I**–**L** Sirius red. Scale bar = 50μm. **M** One day after CCl_4_ administration, it caused similar liver damage in WT, Par2KO, Par2KO, and BM-reconstituted WT and WT BM-reconstituted Par2KO mice. Liver damage at day 1 appeared as clusters of fat vacuoles (**E**, **I**, **G**, and **K** and in Fig. 7G, L, I, N), and clusters were measured as the number of vacuoles within an area of 25,000μm^2^. There is no significant difference between groups in liver damage at day 1, *n*=3–5. **N** Seven days after CCl_4_ treatment, liver damage in Par2KO and WT-reconstituted Par2KO mice was more apparent compared to WT and Par2KO-reconstituted WT mice. Liver damage was measured as the percentage area of necrotized lesions, *n*=5–7. Statistical significance was measured using Mann-Whitney test. *Indicates *p*<0.05, **p<0.01, ***p<0.005, error bars = SEM

Previously, we showed that Par2 is required for tissue regeneration after injury induced by CCl_4_ [37. ]. To extend the observations we presented previously, we repeated the CCl_4_ experiments and completed the histological staining with Sirius red (Fig. 7). In both WT and Par2KO mice, CCl_4_ presented significant liver damage one day after injection (compare WT in Fig. 7B, G, L, to Par2KO in 7D, I, N, quantified in Fig. 8M. Liver markers are presented in Figure S8). To negate the possibility that Par2 is protective against CCl_4_, we conducted TUNEL staining (Figure S9). TUNEL staining indicates that hepatocellular death is evident in both WT and Par2KO mice.

To demonstrate that the immune system does not play a deleterious role in this damaging process, we repeated the BM replacement experiments described above, followed by CCl_4_ treatment (Fig. 8). It is apparent that the damage initiated by CCl_4_ on day 1 was similar in both WT, Par2KO, WT BM-reconstituted Par2KO, and in Par2KO BM-reconstituted WT mice (as indicated by tissue morphology quantification in Fig. 8M). Some of the injury liver markers on day 1 were more significant in the Par2KO and in the WT BM-reconstituted Par2KO mice compared to the WT and Par2KO-reconstituted WT mice (Figure S8). However, in the regenerative response, as seen by liver recovery on day 7, there was a clear hepatocellular recovery advantage in WT (Fig. 7C, H, M compared to Par2KO E, J, O) and in Par2KO BM-reconstituted WT mice (Fig. 8B, F, J, compared to WT BM-reconstituted Par2KO mice D, H, L. All combinations are summarized in Fig. 8N). Therefore, we conclude that Par2 signaling in the immune system had no apparent role in liver regeneration, as WT BM-reconstituted Par2KO mice did not demonstrate any regenerative advantage over the Par2KO.

## Discussion

Inflammation causes many degenerative and chronic conditions, but it also acts to alleviate infections, clear dead cells as well as cell fragments, and initiate tissue recovery. Therefore, manipulating the direction of inflammatory processes beneficially will facilitate faster regenerative responses. Especially when inflammatory processes become too intense or too chronic such as in cases of shock or autoimmunity. The work herein positions Par2 at the “crossroads” between inflammation and regeneration. While recently, there is a wealth of mechanistic studies shedding light on Par2 biochemical processes [29. –36. ], and Par2 function remains perplexing, sometimes worsening an injury while sometimes alleviating it. In this work, we aimed to solve Par2 puzzling role in inflammation and tissue regeneration at the organismal level.

ConA injection and CCl_4_ poisoning are two classical models of toxin-induced hepatitis. The difference between the two experimental systems is that while CCl_4_ is considered a necrotic model in which the hepatocellular tissue degenerates [53. , 58. ], ConA injection is often used to model autoimmune hepatitis [48. , 49. ]. We used the two models in WT and Par2KO mice and showed that in Par2KO mice compared with WT, there was greater hepatocellular damage in the CCl_4_ model [37. ]. In contrast, in the ConA model, Par2KO mice were protected from hepatitis (as defined by mononuclear cell infiltrations, Fig. 1 and S2). These observations were consistent in males and females, with no statistical differences between the sexes.

In the ConA model, Par2 is utilized in the immune system to induce damage that acts as the root cause of the model. In CCl_4_, Par2 in hepatocytes exercises positively to promote regeneration and healing. To distinguish between the hepatic and immune system functions of Par2, we conducted reciprocal BM replacement experiments. We showed that in the ConA model, hematopoietic Par2 was required for ConA-induced inflammation, as demonstrated by the lack of infiltrates and damage when Par2KO BM was used to reconstitute WT mice (Fig. 3F, I).

On the other hand, we showed that hepatic Par2 was required for regeneration in the CCl_4_ experiments (Figs. 7 and 8) and that hematopoietic Par2KO could not protect against hepatocellular necrosis in the WT animals (Fig. 8). Par2 activation could activate hepatocyte regeneration directly and/or could regulate the hepatic stellate cells to inhibit scar formation and induce healthy recovery. Our findings demonstrate that Par2’s function in alleviating or aggravating damage is determined by the role of the immune system in the damage model. If it plays a role in aggravating inflammation, then Par2 activation will have a worsening effect, while if it plays a minor role in the damage model, then Par2 activation in the tissue will elicit regeneration and faster healing.

We noticed that in the ConA experiment (low dose), Par2 expression was retained in hepatocytes adjacent to the immune infiltrates (Figs. 1H, 2, S2B, and S4). We determined that this phenomenon is the result of the inflammatory process and not its cause since Par2KO mice reconstituted with WT BM exhibited infiltrates where Par2 expression in hepatocytes is not possible. It is plausible that adjacent to leukocyte infiltrates, hepatocytes continue to express Par2 at day 14 because different leukocytes secrete proteases, including trypsin [59. , 60. ], and that Par2 activation was proven to stimulate further Par2 expression [61. ].

We define the ConA model with three hallmarked indications: mononuclear cell infiltrations, hemorrhage, and direct hepatocellular damage. In the WT, all these indications appear simultaneously. By using Par2KO, we were able to separate the three processes. We mapped infiltrations and hemorrhage to the immune response, as Par2KO or Par2KO BM-reconstituted WT mice did not present these phenotypes. However, necrosis was observed in all mice, indicating that in addition to recruiting the immune cells, ConA can inflict direct damage to hepatocytes. Here, we identified a protective role of Par2 in hepatocytes, as WT mice did not have necrotic lesions when T cells were depleted, whereas Par2KO mice still exhibited these lesions (compare Figure S7C to E quantified in Fig. 4E). Apart from Par2’s role in promoting regeneration, we found that Par2 has an additional minor protective role in hepatocytes in the CCl_4_ model. While CCl_4_ induces hepatocellular death in both WT and PAR2KO mice (Figure S9), Par2KO and WT BM-reconstituted Par2KO mice have more profound liver injury serum markers on day 1, in comparison to WT and Par2KO BM-reconstituted WT mice (Figure S8B-E). All these phenomena were apparent in both sexes, so we conclude that sex has a minor influence on the Par2 response.

To test the hypothesis that Par2 activation in immune-mediated damage models has an aggravating role, while in regenerative or healing models, Par2 has a beneficiary role, we analyzed the literature reports according to the immune system’s role in the different experimental setups. We looked for immune-mediated as well as direct damage models that were carried out in the same tissue. As we predicted, in immune-mediated damage models, Par2 activation had a deleterious role, whereas in models in which direct damage was induced with little or no involvement of the immune system as the propagator of the damage, Par2 activation induced tissue regeneration, which could be viewed as a protective role (Table 1).

**Table 1:** Par2 modulation experiments conducted in the same tissue assigned according to the observed phenotype. Note that in the cases in which the damage is mediated by the immune system, Par2 has an aggravating role, while in the cases in which the damage is inflicted directly to the tissue, Par2 activation alleviates the damage

**System**	**PAR2 as a phenotypic Alleviator/acts primarily in the tissue**	**PAR2 as a phenotypic aggravator/ acts primarily in the immune system**	**Reference**
Respiratory	Dissected sub-mucosal glands secrete mucus to alleviate injury		[62. ]
		Naive T cells respond to the allergen papain through PAR2, to produce IL-4 and other chemokines.	[63. ]
Colitis and inflammatory bowel diseases	protective effects of PAR2 agonists on HCl/ethanol-induced gastric mucosal injury in WT mice		[8. ]
		Mediates the pro-inflammatory effects of TxA from C. difficile.	[64. ]
Neurological disorders	PAR2KO increases the acute ischemic cerebral injury		[65. ]
		PAR2 modulates neuroinflammation and T cell proliferation during experimental autoimmune encephalomyelitis	[66. ]
Pancreatitis	Ameliorates caerulein-induced pancreatitis		[37. , 67. ]
		Exerts a worsening effect in a bile salts induced pancreatitis	[44. ]

When treating an injury, one needs to consider the roles of the immune system. In cases in which inflammatory processes are deleterious, such as autoimmune diseases, it could be beneficial to inhibit Par2 using pharmacological antagonists. In cases in which tissue regeneration is desired, such as *β* cell neogenesis or digit regeneration that we showed previously to require Par2 activation, specific Par2 agonists may be beneficial.

## Conclusions

ConA injection caused limited damage in Par2KO mice livers, while in WT, severe damage followed by leukocyte infiltrations was evident. Reciprocal BM replacement showed that Par2KO BM reconstitution of WT mice was protective from acute hepatocellular damage, while WT BM reconstitution of Par2KO mice led to a more apparent inflammatory phenotype. In the CCl_4_ direct damage model, WT mice hepatocytes regenerated, while Par2KO mice failed to recover. Reciprocal BM replacements did not show a significant difference in hepatic regeneration compared to the mice that were not transplanted, regardless of the identity of the BM origin. Comparing the models, we conclude that when Par2 is activated in the immune system, it aggravates inflammation and when it is activated in the damaged tissue, it promotes regeneration. When we revisit the different damage models reported in the literature, we resolve Par2’s conflicting roles: If the damage is propagated by inflammation, Par2 activation will aggravate it; If the damage is mediated by direct tissue injury, Par2 activation will induce regeneration.

## Supplementary Information

**Additional file 1: Supplemental Table 1.** Antibodies quantities. **Supplemental Figure 1.** WT and Par2KO mice livers before treatment appear normal. At day 0 there was no difference between WT mouse liver (A, C) to Par2KO mouse liver (B, D) both in macroscopic (A, B) and in microscopic (C, D) appearance. C and D are stained with H&E. Scale bar = 50μm. **Supplemental Figure 2.** ConA induced Par2 expression and mononuclear cell infiltrations throughout the WT liver. A. One day after ConA injection (10mg/kg), Par2 expression increased throughout the liver. Low power (upper panel) and high power (lower panel) views. B. 14 days after ConA injection, mononuclear cell infiltrates had increased and were localized with areas of high Par2 expression. Low power (upper panel) and high power (lower panel) views. Scale bars = 75μm. **Supplemental Figure 3.** While there is an increase in all measured inflammatory markers 6 hours after ConA injection, no difference was found between WT and Par2KO mice. A. TNF-α. B. IL-10. C. INFγ. D. IL-4. (*n*=4 Controls and ConA treated WT, *n*=3 ConA treated Par2KO). No differences in inflammatory markers were found at day 14 between untreated and treated animals. Statistical significance was measured using T-test. *Indicates *p*<0.05, **p<0.01, ***p<0.005, error bars = SEM. **Supplemental Figure 4.** At day 14, hepatic leukocytes do not present Par2 expression. Par2 (green) is co-localized with albumin (white), while leukocytes (marked with CD45 in red) are negative for Par2 staining. A- a low power view, B – a high power view. Scale bar 50μm. **Supplemental Figure 5.** ConA induced hepatic infiltrates are composed mainly from T-lymphocytes. 14 days after ConA injection, infiltrates were observed in both WT BM reconstituted Par2KO (*n*=3 in A) and in WT BM reconstituted WT mice (*n*=3 in B). As in the experiment in the WT mice (figure 6), most of the cells in the infiltrates are T-cells (the CD3 identifier in green). We also used the figure to validate BM reconstitution (as in figure 3). Chimeras of WT CD45.1 that were used as BM donors to CD45.2 (WT and Par2KO, A and B respectively) recipient mice were stained for total CD45 (white) and CD45.1 (red) indicated the presence of successful BM transfer. Scale bar =75μm. C. Quantitative analysis of CD45.1/total CD45 and CD3/total CD45. It may be implied that this analysis shows lower BM reconstitution compared to the FACS analysis presented in figure 3, however, note that there are also more T-cells (green) than CD45+ cells, which is practically impossible. Therefore, we conclude that the FACS analysis is a superior quantitative tool over immunostaining, which is a good qualitative tool to illustrate the different cell types in the infiltrate. Both WT BM reconstituted Par2KO and WT BM reconstituted WT mice showed similar results, so they were pooled together (*n*=6). Error bars= SEM. **Supplemental Figure 6.** T-Lymphocyte depletion in WT and Par2KO mice. CD4 and CD8 lymphocytes were successfully depleted in both WT and Par2KO mice. A-B, E-F. FACS analysis of CD4 and CD8 cells from a control WT non-depleted mice, *n*=4 (A) vs. WT mice treated with anti-CD4 and anti-CD8, *n*=5 (B) showing ~69% depletion of CD4 cells and ~97% depletion of CD8. C-D, E-F. Similar levels of T-cell depletion were observed in Par2KO mice. Statistical significance was measured using T-test. *** indicates *p*<0.005, error bars = SEM. **Supplemental Figure 7.** T-cells depletion protected WT mice from ConA damage. A. Untreated WT mice, B. Non-depleted WT mice 6 hours after high dose of ConA injection (15mg/Kg) – hemorrhage is visible. C. High dose of ConA had no effect in WT mice when T-cells were depleted. D, E. Non-depleted (D) and depleted (E) Par2KO mice were protected from high dose ConA effect 6 hours after ConA injection. Note that necrotic lesions are apparent (quantified in figure 4E). Scale bar = 75 μm. **Supplemental Figure 8.** Liver markers are elevated in Par2KO livers with CCl4 treatment, regardless of Par2 expression in the immune system as measured by VetScan VS2. A. Hemolysis (HEM). B. Alanine aminotransferase (ALT). C. Bile acid (BA). D. Gamma glutamyl transferase (GGT). E. Blood urea nitrogen (BUN); *n*= 3-5 for each group. Statistical significance was measured using T-test; error bars= SEM. Black – between two groups, Red – between untreated WT and all the other groups, Blue – between Par2KO or WT BM reconstituted Par2KO mice and all other groups. **Supplemental Figure 9.** CCl_4_ induces hepatocellular death one day after injection. Hepatic damage at day 1. A. TUNEL staining at day 1. PC –the assy’s internal positive control. nt- no treatment; Scale bar= 100 μm. B. Hepatic damage quantified by TUNEL – Positive area per 1000000 μm2 /Total area was analyzed using ImageJ software; Bars represent mean ± SEM.

## Data Availability

All data generated during and/or analyzed during the current study are available from the corresponding author on reasonable request.

## Abbreviations

BM: Bone marrow
cAMP: Cyclic adenosine monophosphate
cGMP: Cyclic guanosine monophosphate
ConA: Concanavalin A
DMEM: Dulbecco’s modified Eagle’s medium
FACS: Fluorescence-activated cell sorter
FBS: Fetal bovine serum
FoxO6: Forkhead box O6
GPCR: G-protein-coupled receptor
HRP: Horseradish peroxidase
IP: Intraperitoneal injection
IVC: Inferior vena cava
KO: Knockout
MHC: Major histocompatibility complex
NFκB: Nuclear factor kappa-light-chain-enhancer of activated B cells
Par2: Protease-activated receptor 2
PBS: Phosphate-buffered saline
TGF-β: Transforming growth factor beta
TRPV: Transient receptor potential vanilloid
WT: Wild type
