# Supplementary material for: Resolving the conflicts around Par2 opposing roles in regeneration by comparing immune-mediated and toxic-induced injuries
Source: Inflamm Regen. 2022 Nov 29;42:52. doi: 10.1186/s41232-022-00238-2 (PMC9706915; doi:10.1186/s41232-022-00238-2)
Supplement: Supplementary file 1 — Additional file 1: Supplemental Table 1. Antibodies quantities. Supplemental Figure 1. WT and Par2KO mice livers before treatment appear normal. At day 0 there was no difference between WT mouse liver (A, C) to Par2KO mouse liver (B, D) both in macroscopic (A, B) and in microscopic (C, D) appearance. C and D are stained with H&E. Scale bar = 50μm. Supplemental Figure 2. ConA induced Par2 expression and mononuclear cell infiltrations throughout the WT liver. A. One day after ConA injection (10mg/kg), Par2 expression increased throughout the liver. Low power (upper panel) and high power (lower panel) views. B. 14 days after ConA injection, mononuclear cell infiltrates had increased and were localized with areas of high Par2 expression. Low power (upper panel) and high power (lower panel) views. Scale bars = 75μm. Supplemental Figure 3. While there is an increase in all measured inflammatory markers 6 hours after ConA injection, no difference was found between WT and Par2KO mice. A. TNF-α. B. IL-10. C. INFγ. D. IL-4. (n=4 Controls and ConA treated WT, n=3 ConA treated Par2KO). No differences in inflammatory markers were found at day 14 between untreated and treated animals. Statistical significance was measured using T-test. *Indicates p<0.05, **p<0.01, ***p<0.005, error bars = SEM. Supplemental Figure 4. At day 14, hepatic leukocytes do not present Par2 expression. Par2 (green) is co-localized with albumin (white), while leukocytes (marked with CD45 in red) are negative for Par2 staining. A- a low power view, B – a high power view. Scale bar 50μm. Supplemental Figure 5. ConA induced hepatic infiltrates are composed mainly from T-lymphocytes. 14 days after ConA injection, infiltrates were observed in both WT BM reconstituted Par2KO (n=3 in A) and in WT BM reconstituted WT mice (n=3 in B). As in the experiment in the WT mice (figure 6), most of the cells in the infiltrates are T-cells (the CD3 identifier in green). We also used the figure to validate BM reconstitution ( [file 41232_2022_238_MOESM1_ESM.pdf]

CD8 (eFluor 450, Rat, Thermos Fisher Scientific).	1µl
CD4 (Alexa Flour 700, Rat, Thermos Fisher Scientific).	2µl
CD45.1 (PE, Mouse, Thermos Fisher Scientific).	2.5µl
CD45.2 (APC, Mouse, Thermos Fisher Scientific).	2.5µl
CD45 [FITC, Mouse (Isotype recombinant human IgG1), Miltenyi Biotec].	1µl
CD19 [APC-Vio770, Mouse (Isotype recombinant human IgG1), Miltenyi Biotec].	1µl
CD3ε [VioBlue, Mouse (Isotype rat IgG2bκ), Miltenyi Biotec].	1µl
CD4 [PE, Mouse (Isotype recombinant human IgG1), Miltenyi Biotec].	1µl
CD8b [PE-Vio770, Mouse (Isotype recombinant human IgG1), Miltenyi Biotec].	1µl
Anti-F4/80 [APC, Mouse (Isotype recombinant human IgG1), Miltenyi Biotec].	1µl
CD14 [PE-Vio615, mouse (Isotype recombinant human IgG1), Miltenyi Biotec].	1µl

Supplemental table 1. Antibodies quantities.

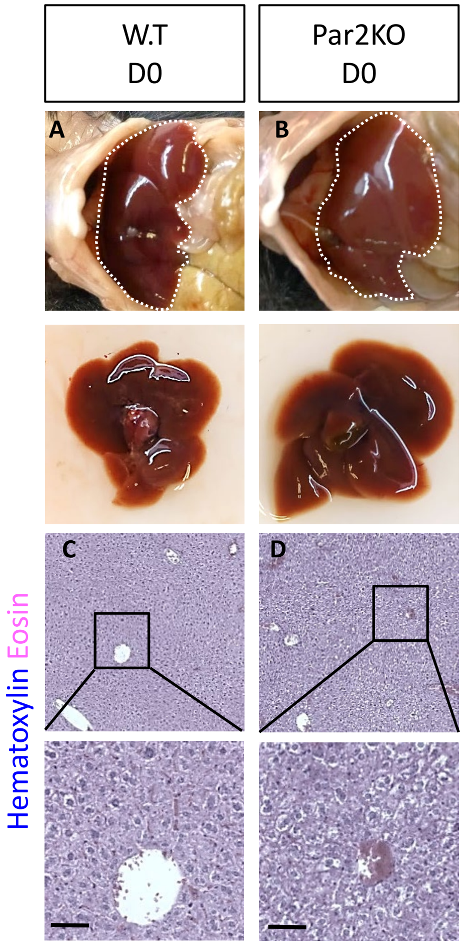

Supplemental figure 1. WT and Par2KO mice livers before treatment appear normal. At day 0 there was no difference between WT mouse liver **(A, C)** to Par2KO mouse liver **(B, D)** both in macroscopic **(A, B)** and in microscopic **(C, D)** appearance. C and D are stained with H&E. Scale bar = 50µm

Reches Supplemental fig. 2

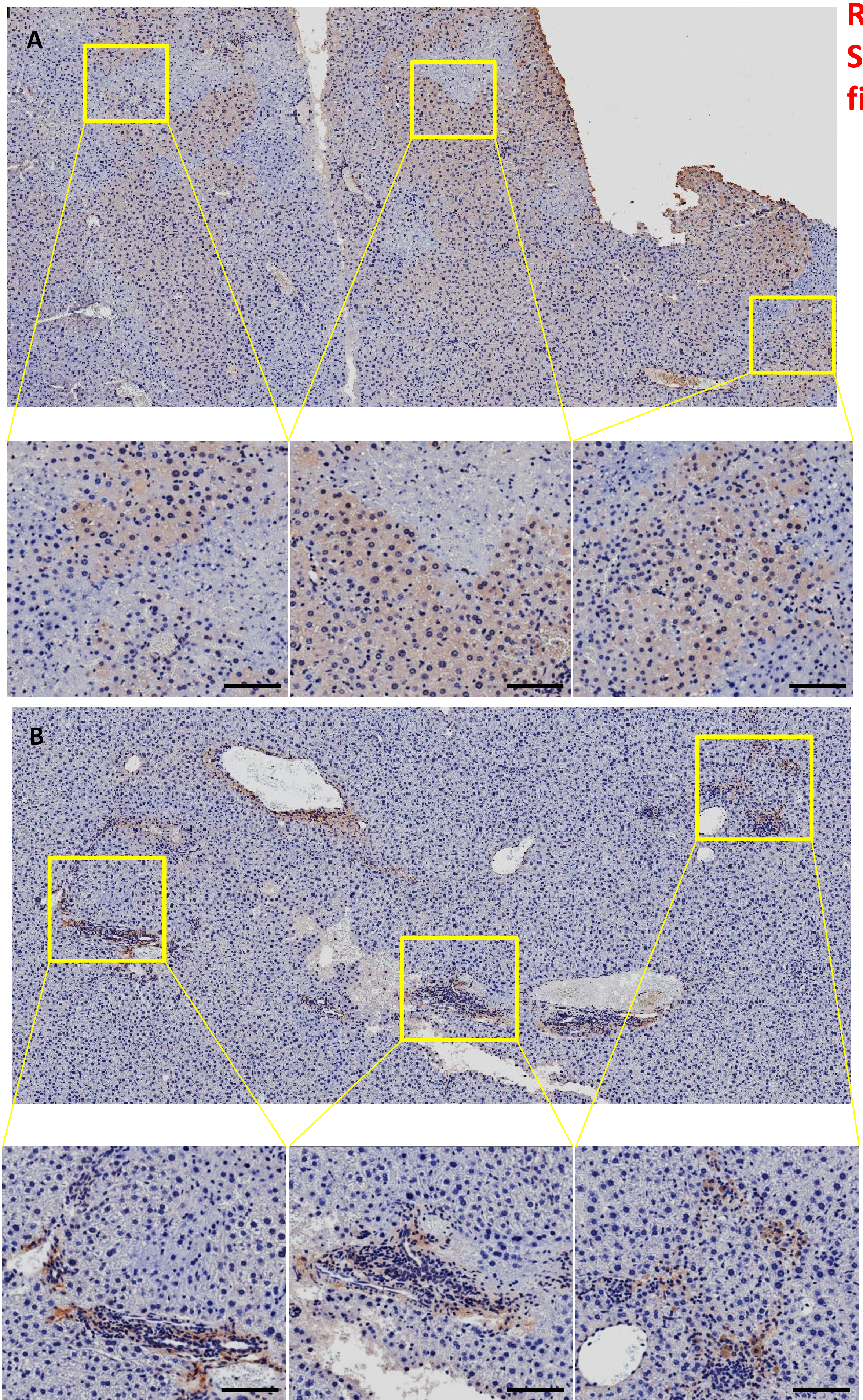

Supplemental figure 2. ConA induced Par2 expression and mononuclear cell infiltrations throughout the WT liver. A. One day after ConA injection (10mg/kg), Par2 expression increased throughout the liver. Low power (upper panel) and high power (lower panel) views. **B.** 14 days after ConA injection, mononuclear cell infiltrates had increased and were localized with areas of high Par2 expression. Low power (upper panel) and high power (lower panel) views. Scale bars = 75µm.

Reches Supplemental fig. 3

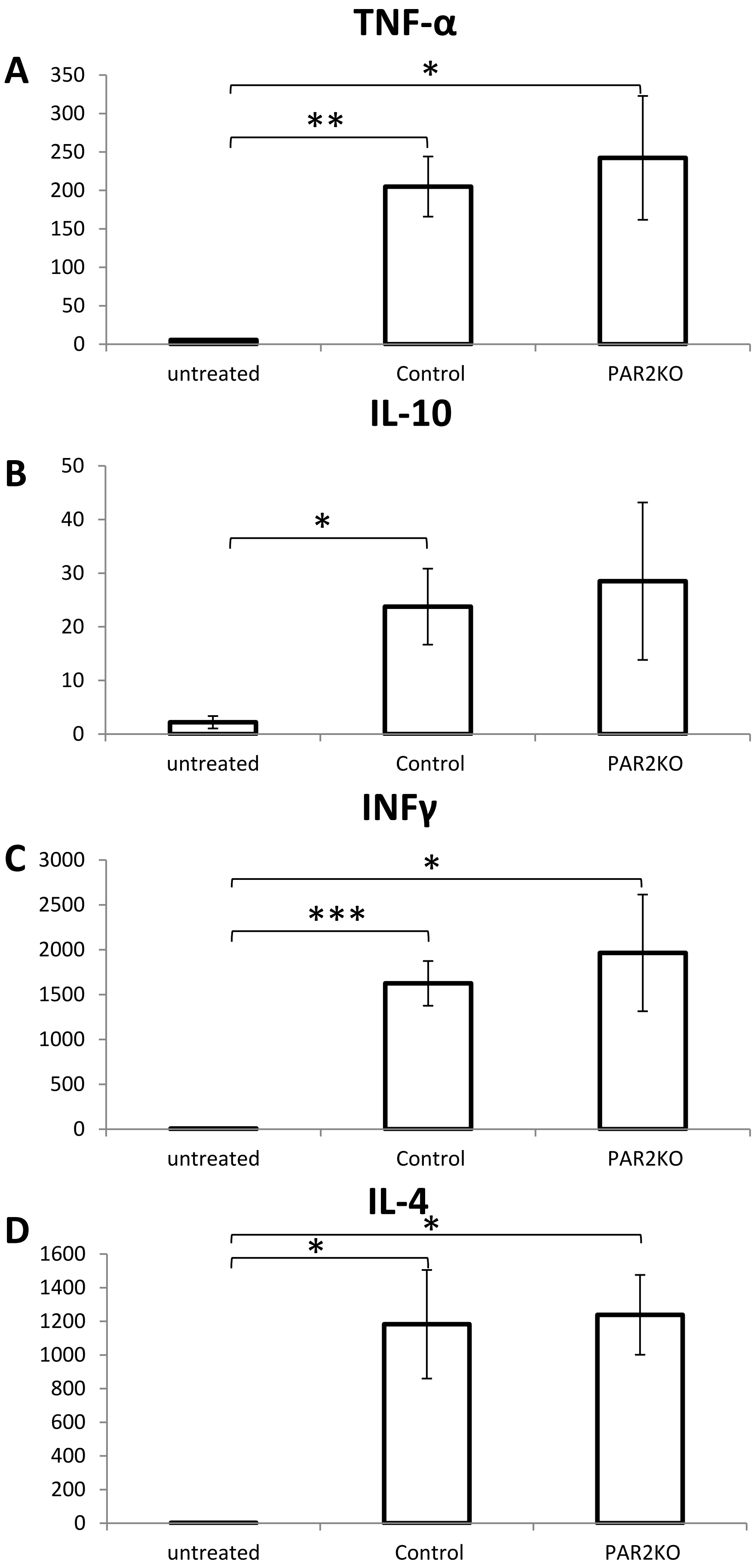

Supplemental figure 3. While there is an increase in all measured inflammatory markers 6 hours after ConA injection, no difference was found between WT and Par2KO mice. A. TNF-α. **B.** IL-10. **C.** INFγ. **D.** IL-4. (n=4 Controls and ConA treated WT, n=3 ConA treated Par2KO). No differences in inflammatory markers were found at day 14 between untreated and treated animals. Statistical significance was measured using T-test * indicates $p<0.05$, ** indicates $p<0.01$ *** indicates $p<0.005$. error bars= SEM.

Reches Supplemental fig. 4

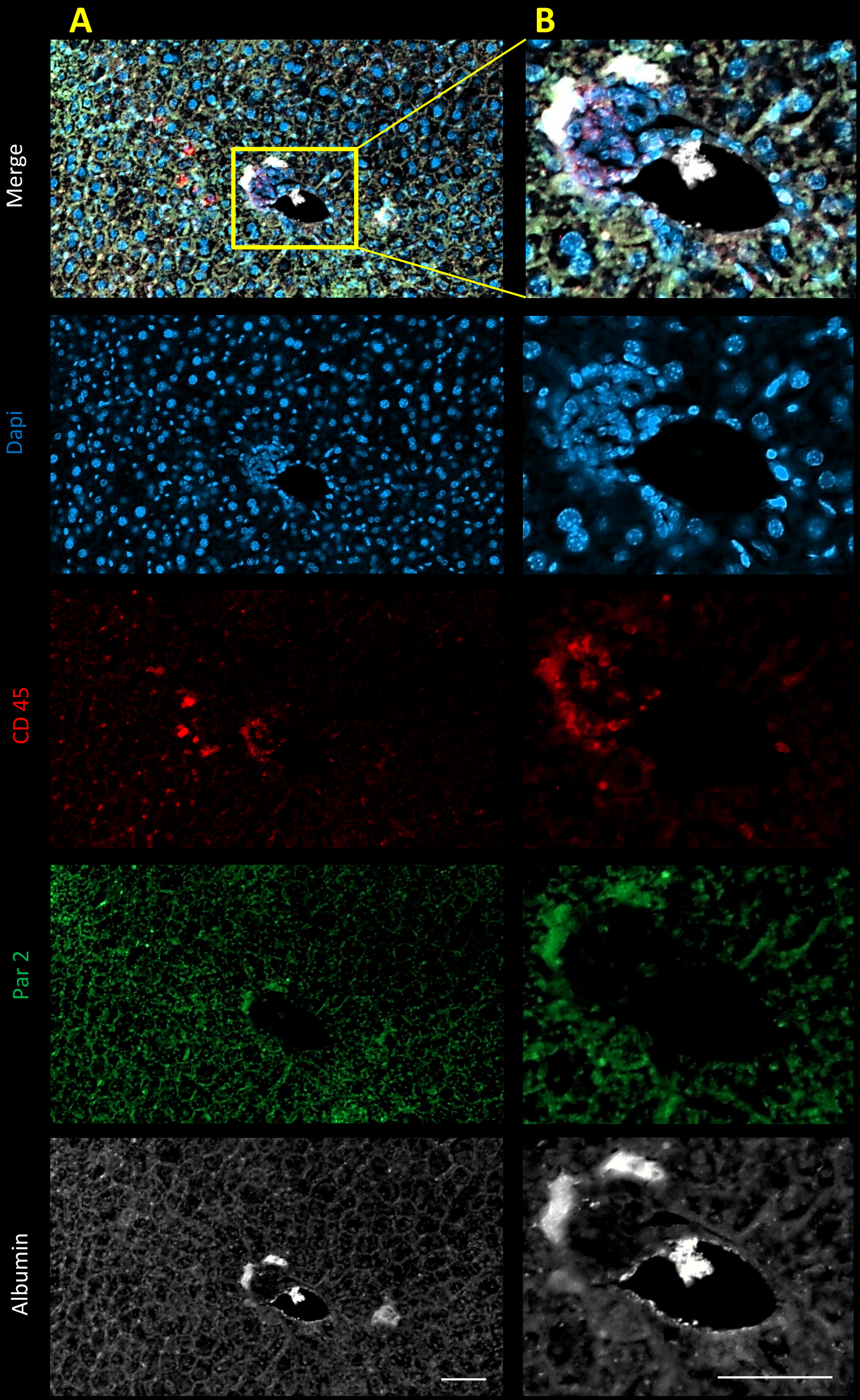

Supplemental figure 4. At day 14, hepatic leukocytes do not present Par2 expression.

Par2 (green) is co-localized with albumin (white), while leukocytes (marked with CD45 in red) are negative for Par2 staining. **A-** a low power view, **B** – a high power view. Scale bar 50μm.

Reches Supplemental fig. 5

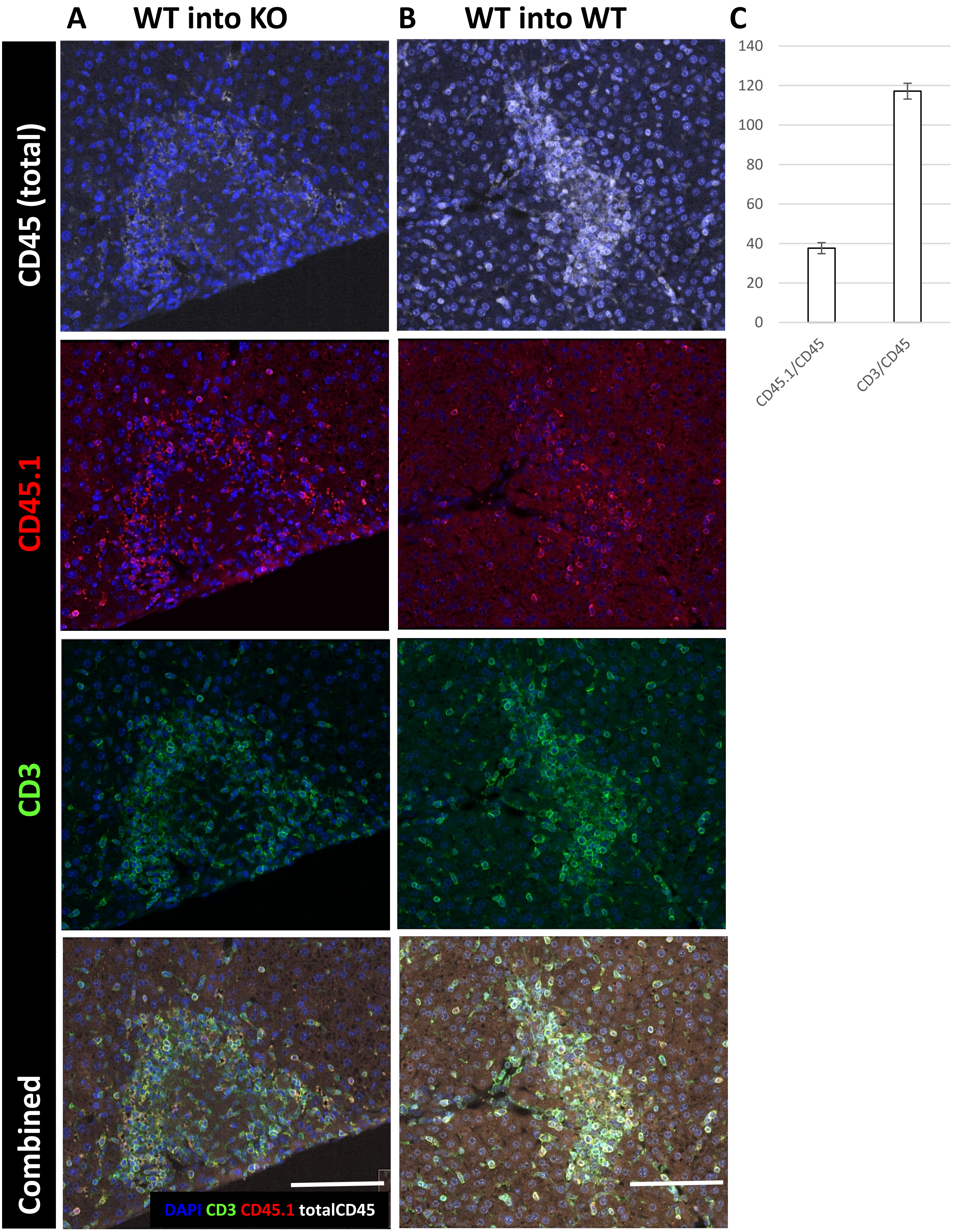

Supplemental figure 5. ConA induced hepatic infiltrates are composed mainly from T-lymphocytes. 14 days after ConA injection, infiltrates were observed in both WT BM reconstituted Par2KO (n=3 in A) and in WT BM reconstituted WT mice (n=3 in B). As in the experiment in the WT mice (figure 6), most of the cells in the infiltrates are T-cells (the CD3 identifier in green). We also used the figure to validate BM reconstitution (as in figure 3). Chimeras of WT CD45.1 that were used as BM donors to CD45.2 (WT and Par2KO, A and B respectively) recipient mice were stained for total CD45 (white) and CD45.1 (red) indicated the presence of successful BM transfer. Scale bar =75µm. **C.** Quantitative analysis of CD45.1/total CD45 and CD3/total CD45. It may be implied that this analysis shows lower BM reconstitution compared to the FACS analysis presented in figure 3, however, note that there are also more T-cells (green) than CD45+ cells, which is practically impossible. Therefore, we conclude that the FACS analysis is a superior quantitative tool over immunostaining, which is a good qualitative tool to illustrate the different cell types in the infiltrate. Both WT BM reconstituted Par2KO and WT BM reconstituted WT mice showed similar results, so they were pooled together (n=6). Error bars= SEM.

Reches Supplemental fig. 6

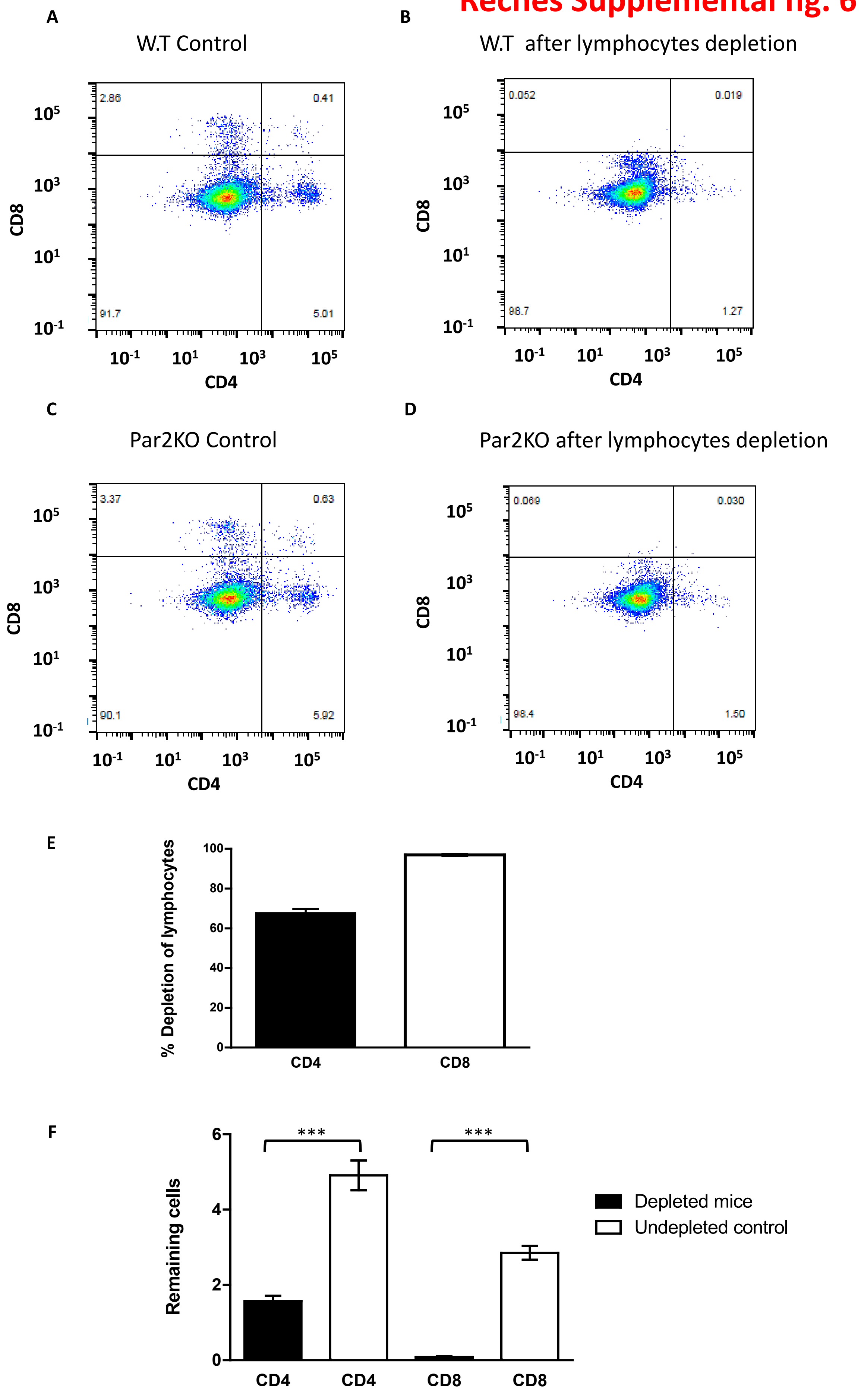

Supplemental figure 6. T-Lymphocyte depletion in WT and Par2KO mice. CD4 and CD8 lymphocytes were successfully depleted in both WT and Par2KO mice. **A-B, E-F.** FACS analysis of CD4 and CD8 cells from a control WT non-depleted mice, n=4 (A) vs. WT mice treated with anti-CD4 and anti-CD8, n=5 (B) showing ~69% depletion of CD4 cells and ~97% depletion of CD8. **C-D, E-F.** Similar levels of T-cell depletion were observed in Par2KO mice. Statistical significance was measured using T-test *** indicates $p<0.005$; error bars= SEM.

Reches Supplemental fig. 7

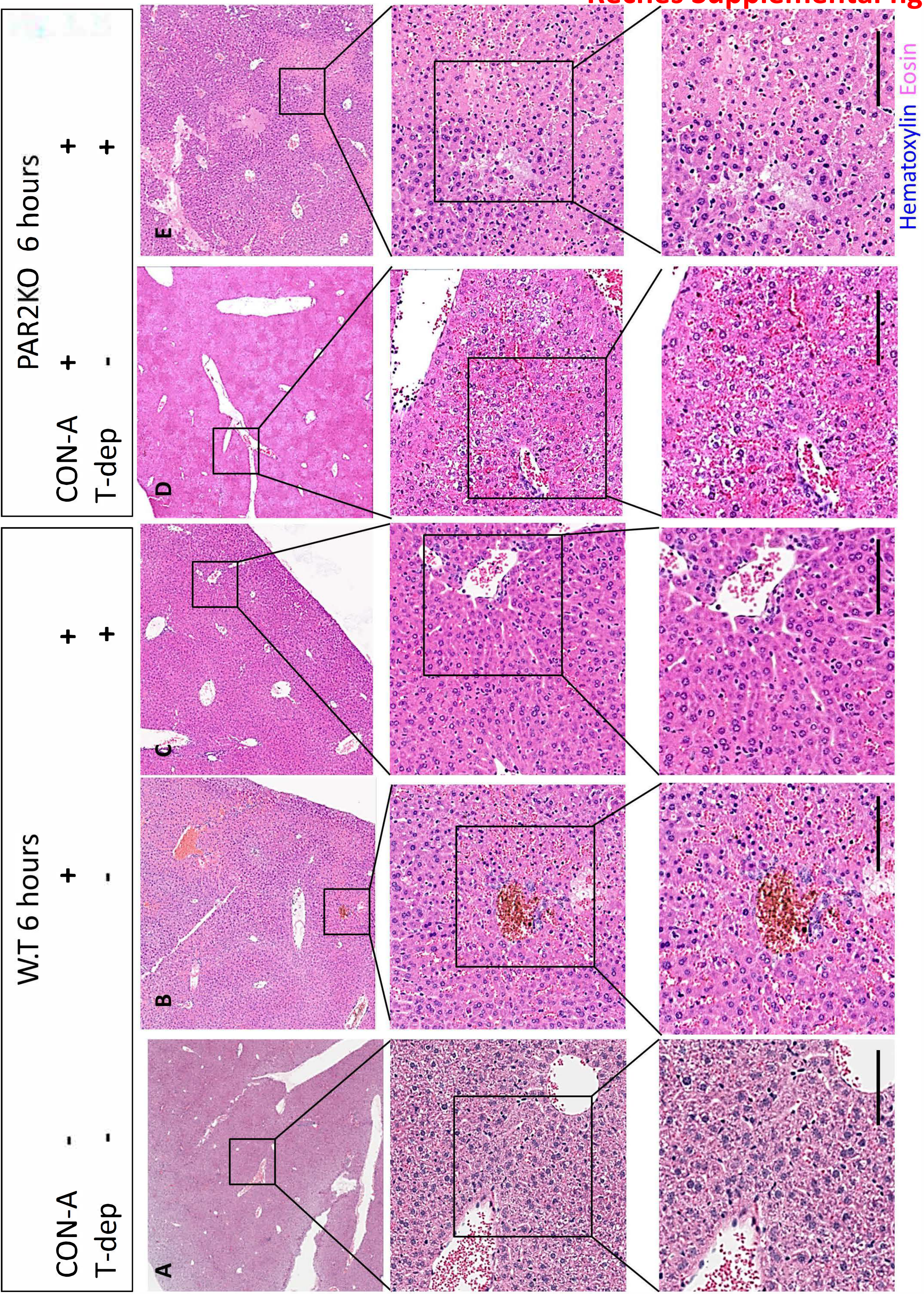

Supplemental figure 7. T-cells depletion protected WT mice from ConA damage. A. Untreated WT mice, **B**. Non-depleted WT mice 6 hours after high dose of ConA injection (15mg/Kg) – hemorrhage is visible. **C.** High dose of ConA had no effect in WT mice when T-cells were depleted. **D, E.** Non-depleted (D) and depleted (E) Par2KO mice were protected from high dose ConA effect 6 hours after ConA injection. Note that necrotic lesions are apparent (quantified in figure 4E). Scale bar = 75 µm.

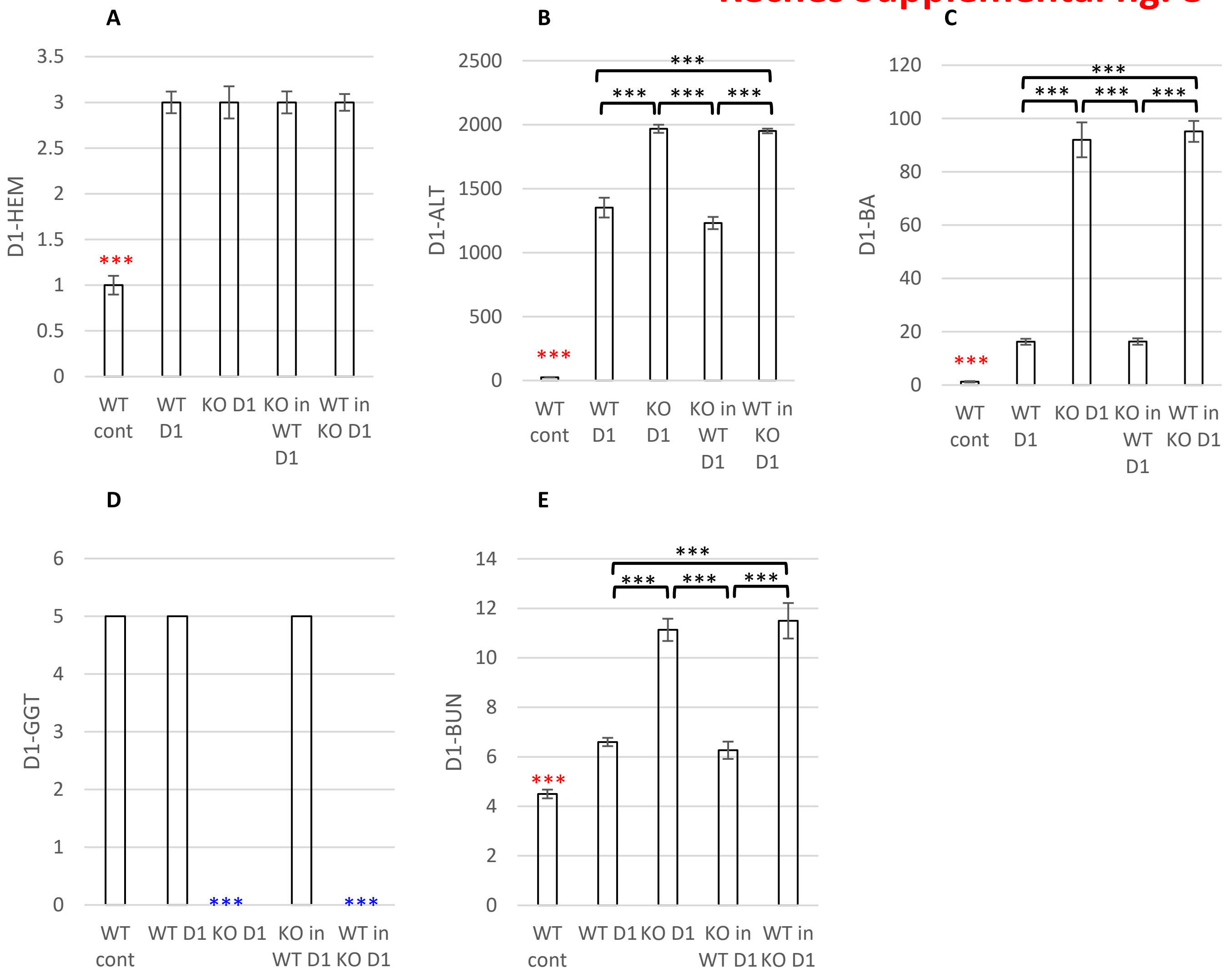

Supplemental figure 8. Liver markers are elevated in Par2KO livers with CCl_4 treatment, regardless of Par2 expression in the immune system as measured by VetScan VS2. A. Hemolysis (HEM). **B.** Alanine aminotransferase (ALT). **C.** Bile acid (BA). **D.** Gamma glutamyl transferase (GGT). **E.** Blood urea nitrogen (BUN); n= 3-5 for each group. Statistical significance was measured using T-test; error bars= SEM. Black – between two groups, Red – between untreated WT and all the other groups, Blue – between Par2KO or WT BM reconstituted Par2KO mice and all other groups.

Reches Supplemental fig. 9

Hepatic damage at day 1

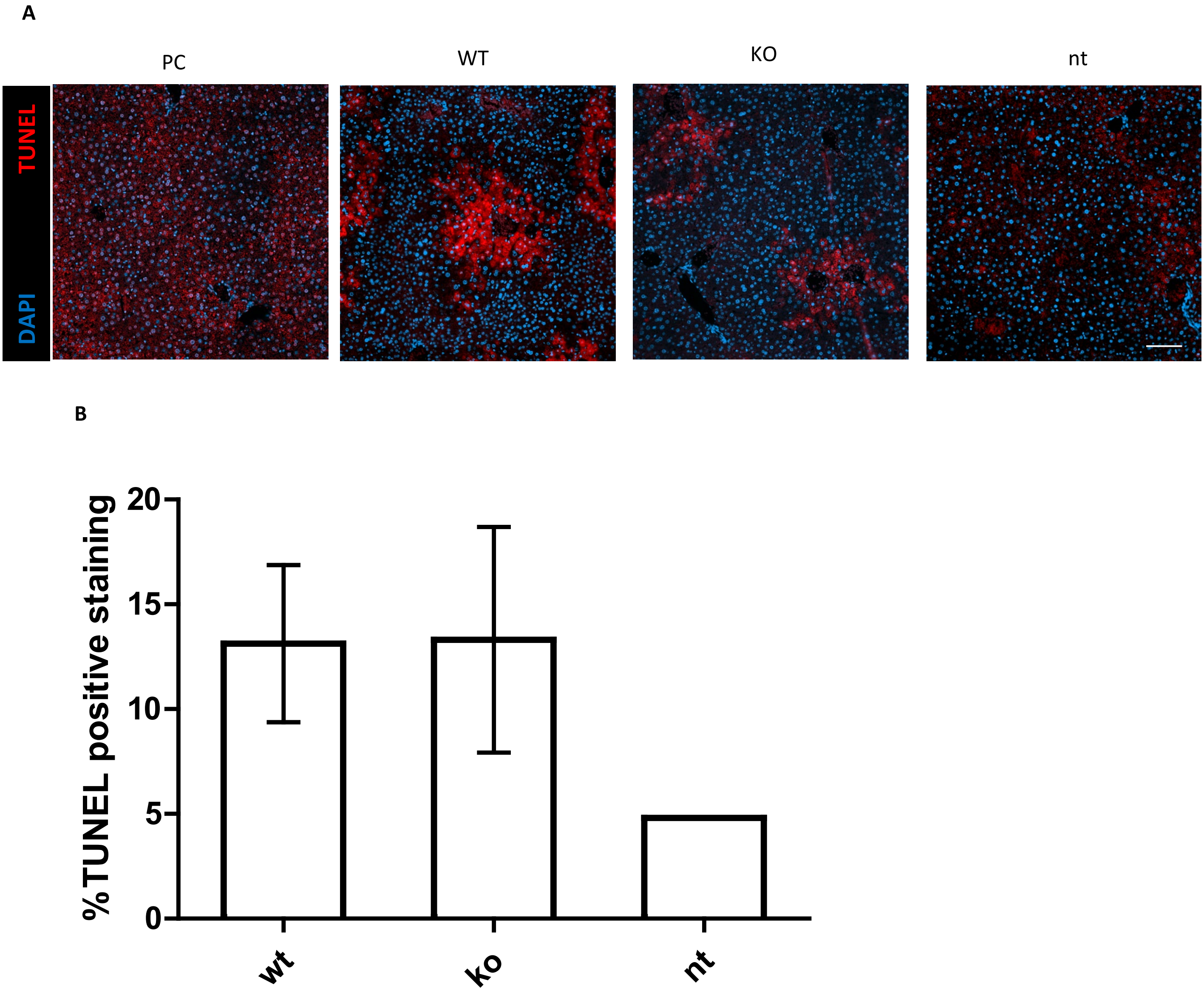

Supplemental figure 9. CCl4 induces hepatocellular death one day after injection.

Hepatic damage at day 1. **A**. TUNEL staining at day 1. PC –the assy's internal positive control. nt- no treatment; Scale bar= 100 µm. **B**. Hepatic damage quantified by TUNEL – Positive area per 1000000 µm^2 /Total area was analyzed using ImageJ software; Bars represent mean ± SEM
